# Family group decision‐making for children at risk of abuse or neglect: A systematic review

**DOI:** 10.1002/cl2.1088

**Published:** 2020-07-12

**Authors:** Tony McGinn, Paul Best, Jason Wilson, Admire Chereni, Mphatso Kamndaya, Aron Shlonsky

**Affiliations:** ^1^ School of Sociology and Applied Social Studies Ulster University Derry/Londonderry UK; ^2^ School of Social Sciences, Education and Social Work Queens University Belfast UK; ^3^ School of Health Sciences Ulster University N. Ireland UK; ^4^ Department of Anthropology and Development Studies Johannesburg South Africa; ^5^ School of Public Health Johannesburg South Africa; ^6^ Department of Social Work Monash University Melbourne Australia

## Abstract

**Background:**

Capturing the scale of child maltreatment is difficult, but few would argue that it is anything less than a global problem which can affect victims’ health and well‐being throughout their life. Systems of detection, investigation and intervention for maltreated children are the subject of continued review and debate.

**Objectives:**

To assess the effectiveness of the formal use of family group decision‐making (FGDM) in terms of child safety, permanence (of child's living situation), child and family well‐being, and client satisfaction with the decision‐making process.

**Search Methods:**

Both published and unpublished manuscripts were considered eligible for this review. Library staff from Scholarly Information (Brownless Biomedical Library) University of Melbourne, conducted 14 systematic bibliographic searches. Reviewers also checked the reference lists of all relevant articles obtained, and reference lists from previously published reviews. Researchers also hand‐searched 10 relevant journals.

**Selection Criteria:**

Study samples of children and young people, aged 0–18 years, who have been the subject of a child maltreatment investigation, were eligible for this review. Studies had to have used random assignment to create treatment and control groups; or, parallel cohorts in which groups were assessed at the same point in time. Any form of FGDM, used in the course of a child maltreatment investigation or service, was considered an eligible intervention if it involved: a concerted effort to convene family, extended family, and community members; and professionals; and involved a planned meeting with the intention of working collaboratively to develop a plan for the safety well‐being of children; with a focus on family‐centred decision‐making.

**Data Collection and Analysis:**

Two review authors independently extracted the necessary data from each study report, using the software application Covidence. Covidence highlighted discrepancies between data extracted by separate reviewers, further analysis was conducted until a consensus was reached on what data were to be included in the review. Two authors also independently conducted analyses of study bias.

**Main Results:**

Eighteen eligible study reports were found, providing findings from 15 studies, involving 18 study samples. Four were randomised controlled trials (RCTs; *N* = 941) the remainder employed quasi‐experimental designs with parallel cohorts. Three of the quasi‐experimental studies used prospective evaluations of nonrandomly assigned comparison groups (*N* = 4,368); the rest analysed pre‐existing survey data, child protection case files or court data (*N* = 91,786). The total number of children studied was 97,095. The longest postintervention follow‐up period was 3 years. Only four studies were conducted outside the United States; two in Canada and one in Sweden and one in the Netherlands. The review authors judged there to be a moderate or high risk of bias, in most of the bias categories considered. Only one study referenced a study protocol. Eleven of the fifteen studies were found to have a high likelihood of selection bias (73%). Baseline imbalance bias was deemed to be unlikely in just two studies, and highly likely in nine (60%). Confounding variables were judged to be highly likely in four studies (27%), and contamination bias was judged highly likely in five studies (33%). Researcher allegiance was rated as a high risk in three studies (20%) where the authors argued for the benefits of FGDM within the article, but without supporting references to an appropriate evidence base. Bias from differential diagnostic activity, and funding source bias, were less evident across the evidence reviewed. This review combines findings for eight FGDM outcome measures. Findings from RCTs were available for four outcomes, but none of these, combined in meta‐analysis or otherwise, were statistically significant. Combining findings from the quasi‐experimental studies provided one statistically significant finding, for the reunification of families, favouring FGDM. Ten effect sizes, from nine quasi‐experimental studies, were synthesised to examine effects on the reunification of children with their family or the effect on maintaining in‐home care; in short, the effect FGDM has on keeping families together. There was a high level of heterogeneity between the studies (*I* ^2^ = 92%). The overall effect, based on the combination of these studies was positive, small, but statistically significant: odds ratio (OR), 1.69 (confidence interval [CI], 1.03, 2.78). Holinshead's (2017) RCT also measured the maintenance on in‐home care and reported a similar result: OR, 1.54 (CI, −0.19, 0.66) not statistically significant. The overall effect for continued maltreatment from meta‐analysis of five quasi‐experimental studies, favoured the FGDM group, but was not statistically significant: OR, 0.73 (CI, 0.48, 1.11). The overall combined effect for continued maltreatment, reported in RCTs, favoured the control group. But it was not statistically significant: OR, 1.29 (CI, 0.85, 1.98). Five effect sizes, from nonrandomised studies, were synthesised to examine the effect of FGDM on the number of kinship placements. The overall positive effect based on the combination of these studies was negligible: OR, 1.31 (CI, 0.94, 1.82). Meta‐analysis was not possible with other outcomes. FGDM's role in expediting case processing and case closures was investigated in six studies, three of which reported findings favouring FGDM, and three which favoured the comparison group. Children's placement stability was reported in two studies: an RCT's findings favoured the control, while a quasi‐experimental study's findings favoured FGDM. Three studies reported findings for service user satisfaction: one had only 30 participants, one reported a statistically significant positive effect for FGDM, the other found no difference between FGDM and a control. Engagement with support services was reported in two studies; neither reported statistically significant findings.

**Authors' Conclusions:**

The current evidence base, in this field, is insufficient to draw conclusions about the effectiveness of FGDM. These models of child protection decision‐making may help bring about better outcomes for children at risk, or they may increase the risk of further maltreatment. Further research of rigour, designed to avoid the potential biases of previous evaluations, is needed.

## 1 PLAIN LANGUAGE SUMMARY

### 1.1 No evidence that family group decision‐making is better, or worse, than conventional child protection procedures

Family group decision‐making is used to make decisions about how best to protect children, and support families. It engages the family, extended family, and people in the community around the family, in these decisions. It features an independent meeting facilitator, private family time away from professionals and the prioritisation of family plans. This review finds that the evidence base supporting this approach is of poor quality with no clear finding that it is any better or worse than conventional approaches.

### 1.2 What is this review about?

Child maltreatment is a global problem which can affect victims' health and well‐being throughout their life. Debate continues as to effective systems of detection, investigation and intervention for maltreated children.

This review assesses the effectiveness of the formal use of family group decision‐making in terms of child safety, permanence (of child's living situation), child and family well‐being, and client satisfaction with the decision‐making process.

**What is the aim of this review?**

This Campbell systematic review assesses the effectiveness of family group decision‐making to tackle child abuse. It summarises the evidence from 15 studies is four countries, with most studies being from the USA.

### 1.3 What studies are included?

The included studies were about children and young people, aged 0‐18 years, who had been the subject of a child maltreatment investigation. Studies had to have used random assignment to create treatment and control groups; or, parallel cohorts, in which groups were assessed at the same point in time. Any form of family group decision‐making used in the course of a child maltreatment investigation or service was considered an eligible intervention if it involved: a concerted effort to convene family, extended family and community members; and professionals; and involved a meeting with the intention of working collaboratively to develop a plan for the safety and well‐being of children; with a focus on family‐centred decision‐making. 

The review authors found 18 eligible study reports, providing findings from 15 studies, involving 18 study samples. Four of the studies were randomised controlled trials.

All but four studies were conducted outside the USA: two in Canada, one in Sweden and one in The Netherlands.

### 1.4 What are the findings of this review?

Overall, there are few if any significant benefits of family group decision‐making compared to conventional treatment, and the quality of the studies in the evidence base is generally poor.

Four randomised controlled trials found no significant effect on continued maltreatment, reunification of children with families or maintenance of in‐home care, engagement with support services and social support.

The quasi‐experimental studies found a statistically significant finding favouring family group decision‐making for the reunification of families, but not for any other outcomes. In all cases, there is considerable variation in effects between studies.

### 1.5 What do the findings of this review mean?

The low quality of the evidence base, with no clear consistent finding of positive effects, is at odds with the support family group decision‐making enjoys in social work practice. However, these findings should not be used to discard the approach, but rather to identify the sources of possible shortcomings in the model whilst strengthening the evidence base.

It is possible that this disconnect is explained by the theoretical appeal of the approach. Failure to fully implement the model may come from a focus on the planning stage but not the implementation of that plan, or that promised family supports are not forthcoming, or from social workers' reluctance to hand decision‐making over to families.

More studies are needed. It is important that study designs take account of the many sources of bias, particularly selection bias, to which studies of this topic are prone.

### 1.6 How up‐to‐date is this review?

The review authors searched for studies published up to June 2019.

## 2 BACKGROUND

### 2.1 Description of the condition

Child abuse or neglect, referred to here as child maltreatment, remains a global problem which can affect victims’ health and well‐being throughout their life. The World Health Organisation describes the range of abuses children may suffer: physical and emotional ill‐treatment, sexual abuse, neglect, and exploitation that results in actual or potential harm to the child's health, development or dignity (WHO, 2016). Capturing the scale of maltreatment, is difficult (UNICEF, 2017) but it is estimated that, worldwide, 41,000 child homicides occur each year and as many as one in four adults were physically abused as children (WHO, 2016). Commonly accepted impacts of childhood maltreatment, during the adult life‐course, include clinical depression and anxiety (Li, D'arcy, & Meng, 2016). Evidence to suggest that child maltreatment is passed on from generation‐to‐generation is complex, but extensive (Bartlett, Kotake, Fauth, & Easterbrooks, 2017) and suggests that the parenting capacity of victims of childhood maltreatment can be reduced.

Statutory intervention on behalf of maltreated children is the responsibility of child welfare agencies in most countries. However, systems of detection, investigation and intervention for maltreated children are the subject of continued review and debate. Historically, the removal of maltreated children from their home to a place of perceived safety and better care, often institutionalised care, was the preferred intervention for statutory welfare services. While removing a child is still necessary in some cases, this action is recognised as less preferable than developing safe care, and parenting, for children in their own home; not least because of research evidence suggesting a lack of stability and poor outcomes for children removed from their home (Wick, 2014). Modern child welfare services therefore endeavour to maintain in‐home care for the children they work with. However, investigations and interventions to address child maltreatment are often hinged upon a dilemma between child rescue (removing a child from their home) and supporting a family to maintain a child safely at home (Rauktis, McCarthy, Krackhardt, & Cahalane, 2010).

The United Nations Convention on the Rights of the Child (UNICEF, 1989) promoted more involvement of children in decisions regarding their care. Subsequent decades also saw a focus on developing family participation in decision‐making, and planning, in order to keep children safe within their own family (Pennell & Burford, 2000). Developing mutual relationships between families, children and professionals became a widely accepted priority in child protection work (Berzin, 2006). While the progression of child welfare policy and practice has taken a number of twists and turns over the past 40 years, and differs considerably across regions, there has clearly been a shift towards sharing power and problem‐solving with families (Frost, Abram, & Burgess, 2014; Rauktis et al., 2010).

Against this background of progress in children's and family rights and, also, due in no small measure to indigenous Maori traditions, FGDM emerged in New Zealand as a promising means of formalising family involvement in child protection decision‐making, and forward planning. It became mandatory practice in New Zealand's Children, Young Persons, and Their Families Act, 1989, and child protection policy makers across the globe have been implementing or trialling versions of FGDM since (D. Crampton, 2007; Merkel‐Holguin, Nixon, & Burford, 2003).

### 2.2 Description of the intervention

In 2007 Crampton suggested that, worldwide, approximately 150 communities had explored, or were exploring, the benefits of FGDM in child welfare practice. More recently, Frost et al. (2014) suggested that the use of FGDM was increasing in child protection work. Acknowledging that FGDM initiatives are on‐going in parts of Europe, the United States, Canada and Australia, we would also point out that it is not main‐stream child protection practice outside New Zealand. The prominence of FGDM varies regionally and, we would add, temporally as policy makers focus and refocus on the various objectives of child protection work.

Several FGDM practice manuals have been published (Jones & Daly, 2004; Partnership for Strong Families, 2009). Berzin's (2008, p. 36) summary captures the central tenets of the intervention:

“In general terms, FGDM is a child welfare decision‐making process in which efforts are made to bring together all parties with an interest in the well‐being of the child and his or her family. At the FGDM meeting, the group works to discuss the concerns that bring the child to the attention of protective services, the strengths that exist in the family system, and the changes necessary to keep the child safe”.

Proponents see it as a means of empowering families to problem solve, a means of enhancing parenting and care capacity to help protect against child maltreatment, and a better conduit to securing permanency of parenting and care, than traditional child protection services (Barnsdale & Walker, 2007; Morris and Connolly, 2012; Rauktis et al., 2010).

Whilst the ethos, and most of the key features of FGDM are consistent where ever it is deployed, several versions of FGDM have emerged since the formal documentation of New Zealand's Family Group Conferencing (FGC) model in 1989 (D. Crampton, 2007). These include: Team Decision Making, Family Team Conferencing, Family Team Meetings, Expedited Family Group Conferencing and Ohana Conferencing. We should also acknowledge that there are variations in the application of these models, regionally, and across agencies (Morris & Connolly, 2012). FGC is arguably the touch‐stone for all FGDM models, and the following intervention components were found to be common to a majority of descriptions offered by previous commentators and researchers (Berzin, Thomas, & Cohen, 2007; D. Crampton, 2007; Dijkstra, Creemers, Asscher, Deković, and Stams, 2016; Doolan, 2007; Hollinshead, 2017; Kempe Centre for Prevention of Child Abuse, 2019; Marsh & Crow, 1998; Merkel‐Holguin, 2003; Partnership for Strong Families, 2009; Rauktis, Bishop‐Fitzpatrick, Jung, & Pennell, 2013; Sundell & Vinnerljung, 2004):

1. Efforts are made to involve the wider family and/or appropriate individuals from the family's community.
2. An independent (i.e., noncase carrying) coordinator chairs one or more meetings of family members and child protection service staff.
3. Family groups are given time to themselves, during, after or between meetings, to help facilitate their own decision‐making and, where appropriate, agreement on a safety plan going forward.
4. Child protection services prioritise family group plans, providing child protection concerns are adequately addressed.

It should be noted that FGDM interventions are an on‐going active process, during the course of a child protection case. Plans agreed by the family group and child protection staff are reviewed at appropriate intervals. They also hinge on child protection agencies providing appropriate support, in line with the plan agreed by the family group, on an on‐going basis. However, these criteria are equally applicable to most non‐FGDM practice models in the child protection domain, and were not used as selection criteria in this review.

Studies which compared FGDM with more traditional child protection services were included in this review. Traditional child protection services are defined here as those in which decision‐making on children's care plans and placement have been professionally driven, with workers conducting assessments of families’ problems and risk profiles, and determining a care plan with which families are asked to comply (Merkel‐Holguin, 2003; Rockhill 1999). Policy and practice guidance in most developed nations now acknowledges the importance of in‐depth engagement with children's families and extended families where possible (Connolly, 2006; Littell, 2001; Yatchmenoff Diane, 2005). However, FGDM remains distinct from traditional services. None of the comparators in the primary studies reviewed here employed independent, FGDM‐trained chairs, for meetings with private family time and a prioritisation of family‐proposed plans.

### 2.3 How the intervention might work

We can explore the hypothesised mechanisms of FGDM in more detail. First, in relation to family empowerment: studies which have researched family's perspectives on FGDM have found that they prefer it to traditional child protection practice (Berzin, 2007). This may be the case because it offers a better balance of power to families, or it may be due to increased family unity, brought about by the FGDM process (Pennell & Burford, 2000). Sundell & Vinnerljung (2004) suggests that families are more likely to accept and buy into a plan that they themselves have proposed, than a plan imposed upon them by professionals.

Second, FGDM is designed to seek out and encourage the participation of extended family and community resources. In this way, FGDM models aim to strengthen the family and community network. Creating a strong network of support around the child and caregiver(s) may improve outcomes for children. Attracting investment from extended family through FGDM is thought to increase the likelihood of a kinship placement, when children must move from their home. The involvement of extended family is also thought to increase the likelihood that, when placed, children will remain with their siblings (Connolly & MacKenzie, 1998; C. Lupton & Nixon, 1999; Marsh & Crow, 1998).

Third, FGDM models frame families as competent and often explicitly focus on their strengths, with the aim of empowering families and shifting their experience of child protection service from one characterised by powerlessness to one of self‐determination and collaboration (C. Lupton & Nixon, 1999). Literature across disciplines indicates that therapeutic settings which support clients’ sense of autonomy, relatedness and competence are more likely to bring about compliance with treatment, and greater transfer and maintenance of treatment gains (Dwyer, Hornsey, Smith, Oei, & Dingle, 2011; Ryan, Lynch, Vansteenkiste, & Deci, 2011).

Finally, on how FGDM might work to improve outcomes for maltreated children we should also reference FGDM's theoretical underpinning: FGDM could be said to align with ecological system theory, social network theory and strengths‐based therapeutic practice and intervention (Havnen & Christiansen, 2014; Morris & Connolly, 2012; Nyberg, 2003). Through concerted engagement with families, FGDM is thought to encourage a more comprehensive trawl of the systems within which a child at risk exists, and is therefore more likely to find intrinsic family strengths which can lead to better outcomes.

### 2.4 Why it is important to do this review

This review contributes to the literature by including the most recent research on FGDM, including outcomes that have not been included in prior reviews, and employing stringent criteria for search, selection, coding, and analysis as specified in the Campbell Collaboration guidelines (Campbell Collaboration, 2016). The question of how effective FGDM is in meeting its objectives has attracted considerable commentary. While commentary on the implementation and success of FGDM is extensive, relatively few studies of efficacy have been conducted (Frost et al., 2014). The current evidence base is routinely cited as positive by researchers studying FGDM, together with reviewers, and commentators on the topic area (including but not limited to: Baumann, 2006; Berzin, 2006; Burford, 1999; Lambert, Johnson, & Wang, 2017; Pennell, Edwards, & Burford, 2010; Sheets et al., 2009).

In 2003, the American Humane Society (2019) published a special issue on FGDM, with 29 submissions from the United States and beyond (forwarded by Merkel‐Holguin, 2003). Much of the material brought together in this special issue relates to the implementation of FGDM projects. Only one of these articles provided sufficient outcome data to facilitate inclusion in the current review. However, Merkel‐Holguin et al. (2003) provided the forward to this volume and summarised that FGDM compared favourably to traditional child protection methods in providing child safety; encouraging kinship placements; encouraging stable children's placements; bringing about reunification with parents, timely decision‐making, increased family support and a reduction in family violence.

Crampton (2006) provided a narrative review of four FGDM evaluations. Primary study effect sizes are not offered. Crampton describes the positive evaluations of two studies (Crampton, 2003; Pennell & Burford, 2000) and the inconclusive findings of Sundell and Vinnerljing's (2004) study and a study by the Centre for Social Services Research (2004). Frost et al. (2014) also completed a narrative review and reported mixed results, while also offering encouragement for the continued practice of FGDM. Frost et al. suggest that studies by Crampton and Jackson (2007); and Pennell and Burford (2000) provide positive results; whilst Berzin (2006) and Sundell and Vinnerljung (2004) provide neutral findings. Frost et al. go on to describe how service users’ evaluations of the process of FGDM are overwhelmingly positive. Participants feel they are being listened to and valued. Frost et al. argue that this demonstrates the value of FGDM in the absence of powerful outcome evidence.

It can be seen that policy makers may find encouragement to deploy FGDM, in these reviews. However, a counter‐standpoint also exists. A number of researchers in this field have argued that the body of evidence supporting FGDM lacks rigour, and that there is insufficient evidence available to make a judgement on the efficacy of FGDM (e.g., Creemers et al., 2016; Havnen & Christiansen, 2014; Weigensberg, Barth, & Guo, 2009). Havnen and Christiansen (2014) found that seven out of ten studies, retrieved for their review, reported positive results. The other three were negative or neutral, and only two of the studies that reported positive results (Wang et al., 2012; Weigensberg et al., 2009) used satisfactory methods. Dijkstra et al. (2016) review is arguably the most comprehensive and rigorous review to date. Dijkstra et al. reviewed 14 studies and concluded that, according to the evidence available, FGDM did not significantly reduce child maltreatment. They highlighted the need for more robust studies of efficacy.

Dijkstra et al. (2016) review has been a step forward in review methodology for the field. Previous reviews did not report systematic literature searches and did not review all of the studies available, or deploy meta‐analysis. However, three studies were included in Dijkstra et al.'s review did not meet the selection criteria for the current review, and an additional three eligible studies were found for the current review.

More generally, there remains a lack of emphasis on study rigour, or a formal assessment of potential bias across FGDM evaluations. In the context of disagreement about FGDM efficacy, a systematic review, completed according to the Campbell Collaboration's standard of methodological rigour (Campbell Collaboration, 2016) provides a more definitive answer to the question. In addition, the acceptance, rejection and discussion of study methodologies, a central focus of Campbell reviews, will provide guidance for the development of more rigorous study protocols, going forward.

In summary, this review considers the problem of how to go about optimum decision‐making for the protection of children from abuse and neglect. This problem is located within on‐going efforts to protect children while also promoting family unity, upholding family's rights and guarding against oppressive statutory intervention in family life. FGDM has been proposed as an effective response to this problem, and this review will help guide the development of this intervention and its evaluation.

## 3 OBJECTIVES

To assess the effectiveness of the formal use of FGDM in terms of child safety, permanence (of child's living situation), child and family well‐being and client satisfaction with the decision‐making process.

## 4 METHODS

### 4.1 Criteria for considering studies for this review

#### 4.1.1 Types of studies

Studies will be eligible for this review if they (a) used random assignment to create treatment and comparison or control groups; or (b) used parallel cohort designs in which groups were assessed at the same points in time (i.e., quasi‐experimental designs that include groups assessed at the same time as opposed to a historical cohort). Single‐group designs and single‐subject designs will be excluded (see “risk of bias” section for further details on included designs).

#### 4.1.2 Types of participants

Children and young people aged 0–18 years who have been the subject of a child maltreatment investigation.

#### 4.1.3 Types of interventions

Any form of FGDM used in the course of a child maltreatment investigation or during the course of services arising from such an investigation. FGDM involves convening family and child protection professionals with one or more of other professionals, extended family, identified friends and/or community members. In an effort to collaboratively develop a plan to maintain child safety, facilitate stable and permanent living arrangements, and promote child well‐being. Therefore, studies will be included in the review if they involve: (a) a concerted effort to convene family, including extended family, friends and community members; and (b) child protection professionals (as well as other professional service providers) participating in; (c) one or more planned meetings with the intention of working collaboratively to develop a plan for the safety, permanence and well‐being of children; (d) with a focus on family‐centred decision‐making; (e) an independent meeting facilitator; (f) private family time during the process.

#### 4.1.4 Types of outcome measures

##### Primary outcomes

Official reports found in administrative data and case files, were the preferred indicators of outcomes, but studies were also accepted if they used standardised recording tools for study participant reports.

The prevention of child maltreatment, and the stability of child placements following the involvement of a child protection service, were the primary outcomes of interest. The success of FGDM, in preventing child maltreatment, was measured by (in order of preference): substantiated or verified referrals to a child protection authority; referrals (with or without substantiation) to a child protection authority; parent‐report; and child self‐report. Indicators of child placement stability differed depending on the childrens' circumstances. If children resided in the homes of their permanent carers, then a move to an out‐of‐home placement was a negative outcome. Therefore, more child removals, in comparison with a non‐FGDM group of children, were indicative of poor efficacy. Kinship placements (placement in out‐of‐home care with relatives) were interpreted as a positive outcome in comparison to other out‐of‐home placements (e.g., residential care). The achievement of legal permanence, for childrens' placements was accepted as a positive outcome. For example, reunification with birth parents, adoption by related or nonrelated caregivers, placement with relative caregivers, legal guardianship/legal custody by related or nonrelated caregivers.

Studies were only included, in the analysis of primary outcomes, if subjects were followed for at least 6 months after the intervention; to allow for sufficient time to observe outcomes. Where outcomes were reported at multiple time points the longest follow‐up period was used in the data synthesis.

##### Secondary outcomes

Secondary outcomes included child well‐being, and client satisfaction with the FGDM process and plan. Data were not excluded on the basis of the validity or reliability of any instruments used. However, the reviewers judgements on the validity and reliability of instruments used formed the basis of their judgement on potential bias due to insensitive measurement instruments.

### 4.2 Search methods for identification of studies

The primary systematic literature search was carried out in July 2016 by library staff, Tania Celeste and Frances Morrissey, from Scholarly Information, University of Melbourne. As this review was in process for 3 years the searches were repeated in August 2019 by the first and second author, date limited from 2016 to 2019. Both published and unpublished were considered eligible for the review. Searches were not restricted to any single language or nationality. One article required translation from Dutch to English, this was completed using Google translate.

#### 4.2.1 Electronic searches

Electronic searches for the identification of appropriate studies were completed as follows:

ASSIA ProQuest Search Strategy (21 July 2016; 2 August 2019)
IBSS ProQuest Search Strategy (22 July 2016; 2 August 2019)
NCJRS ProQuest Search Strategy (22 July 2016; 2 August 2019)
Sociological Abstracts ProQuest Search Strategy (21 July 2016; 2 August 2019)
ProQuest Dissertations & Theses Global Search Strategy (21 July 2016; 2 August 2019)
Family INFORMIT Search Strategy (20 July 2016; 2 August 2019)
CINAHL database using the EbscoHost platform: Search Strategy (20 July 2016; 2 August 2019)
ERIC using the EbscoHost platform: Search Strategy (20 July 2016; 5 August 2019)
SocIndex using the EbscoHost platform: Search Strategy (20 July 2016; 2 August 2019)
Medline using the OVID platform: Search Strategy (14 July 2016; 2 August 2019)
EMBASE searched via the OVID platform (20 July 2016; 5 August 2019)
PsycINFO searched using the OVID platform (14 July 2016; 5 August 2019)
Evidence Based Medicine Reviews was searched via the OVID platform on (14 July 2016; 5 August 2019).

The searches were broadly and substantively similar but leveraged controlled vocabularies and search operators unique to each resource. For example, the construction “random* control* trial” could not be used in ProQuest as the internal wildcards were not recognised. Search facilities were chosen with reference to recent research (McGinn, Taylor, McColgan, & McQuilkan, 2014) on their comparative usefulness for questions related to social work. The search terms, formulae and syntax used on each search facility are described in Appendix C.

#### 4.2.2 Searching other resources

Reviewers checked the reference lists of all relevant articles obtained, and reference lists from previously published reviews. Authors of papers which could potentially have been included in the review, had they reported more details of findings, were emailed. The review team also searched ClinicalTrials.gov and the World Health Organisation's International Clinical Trials Registry Platform.

The following journals were hand‐searched (online) by the review team:

(1) *Child Welfare*
(2) *Children and Youth Services Review*
(3) *Social Service Review*
(4) *Child Maltreatment*
(5) *Child Abuse and Neglect*
(6) *Journal of Social Services Research*
(7) *Social Work*
(8) *Research on Social Work Practice*
(9) *Social Work Research*
(10) *Child Abuse Review*

The following sources of grey literature were searched by the review authors: Social Care Institute for Excellence, the Latin American and Caribbean Centre on Health Sciences Research Institute, and the American Institutes for Research. Several country's government websites were searched: Research at Home Office (UK); U.S. Department of Justice; and the Canadian, Australian, New Zealand, French and German government websites. The Proquest Dissertation and Thesis facility was searched by the University of Melbourne Library Team.

Personal communications were also deployed in the search for relevant articles, as described in the review protocol (Shlonsky et al., 2009) these comprised of face‐to‐face discussions with presenters and emails to experts, and relevant study authors.

### 4.3 Data collection and analysis

#### 4.3.1 Selection of studies

The search outputs, titles and abstracts for 1,576 papers, were uploaded to the software application Covidence. Covidence facilitated the screening and categorisation of the search outputs. Each article title was independently screened by two reviewers. Authors accessed manuscript abstracts, and whole texts where necessary. Covidence facilitates the screening process with a clear audit trail. After duplicates were removed initial screening, by two authors, excluded 1,419 manuscripts. The initial screening questions were: is the population of children and youth who are, or have been, the subject of child protection investigation?; and, is there an intervention related to family group conferencing in the study? Following initial screening, the full text of 100 articles were then independently assessed by two authors against the inclusion and exclusion criteria outlined in the study protocol (Shlonsky et al., 2009). At this level of screening, studies had to satisfy the following criteria: the study evaluated an intervention administered to children and youth aged 0–18; it used an experimental or parallel cohort research design, with a valid control or comparison group. The fundamentals of FGDM, as outlined in section (description of the intervention) were used to ensure study interventions were part of the FGDM family of interventions. Thirteen studies (reported in 15 manuscripts) from the main searches, were found to match selection criteria. Three additional study reports were located through correspondence with primary study authors. One of these provided additional findings for one of the studies located in the main searches; the other two were added to the primary studies for the review, following independent appraisal by two authors. In summary, 15 studies, reported in 18 study reports were selected for review.

#### 4.3.2 Data extraction and management

Two of three review authors, Tony McGinn, Mphatso Kamndaya and Admire Chereni, independently extracted the necessary data from each study report using Covidence. Covidence facilitates the recording of data for:

(1) Study author(s); year of publication; source; country; and language.
(2) Characteristics of setting and participants: eligibility criteria for participants; explanation of recruitment procedures, setting (country, location, clinical/nonclinical); demographic features of the sample.
(3) Sampling: sample sizes for treatment and control; whether power analysis was used to determine sample size; allocation to the treatment and control; explanation of method used to generate the allocation.
(4) Research design: type of design including major features such as random selection, random assignment, and data relating to potential biases.
(5) Intervention data: the nature of the interventions (for treatment and comparison/control groups); FGC, FUM, or some other form of FGDM; aim of intervention; length of intervention, whether manuals were used, whether fidelity checks were included, information on possible contamination reported.
(6) Outcome data: primary and secondary outcomes, measures used, information on reliability/validity of measures.
(7) Results: attrition at postintervention and follow‐up; number excluded from the analysis; length of follow‐up; statistical methods; type of data effect size is based on; data needed for effect size calculations.

Covidence highlights discrepancies between data extracted by separate reviewers, and prompts further analysis of studies until a consensus can be reached on what data is to be included in the review.

#### 4.3.3 Assessment of risk of bias in included studies

Two authors independently conducted analyses under each of the potential bias categories described in Cochrane Collaboration guidance (Higgins et al., 2011):

- Sequence generation
- Allocation sequence concealment
- Blinding of participants and personnel
- Blinding of outcome assessment
- Incomplete outcome data
- Selective outcome reporting
- Other biases.

We considered “other biases” as listed in Cochrane guidance (Higgins et al., 2011):

- Design
- Baseline imbalance
- Differential diagnostic activity
- Insensitive instrument used to measure.

In addition, under “other biases” we considered “researcher allegiance bias” and “funding source bias” for similar reasons as those outlined in Maynard, Solis, Miller, and Brendel (2017): studies are more likely to be biased in favour of the treatment intervention when study authors have a direct role in the development or the implementation of the study. We also considered “contamination bias” as this was highlighted as a possible bias in the review protocol (Shlonsky et al., 2009); we also considered potentially confounding variables, in the study environment (Sterne et al., 2016).

The review authors agreed on a priori guidance for the rating of bias in each primary study (see Appendix A). Each study was categorised as “low”, “high”, or “unclear” risk of bias on each of the domains. Extracts, from primary studies, which might underpin judgements on bias, were compiled and reviewed by two review authors. Any discrepancies between review author judgements were resolved through discussion with a third member of the team.

#### 4.3.4 Measures of treatment effect

##### Continuous data

A standardised mean difference (SMD) was calculated for studies reporting continuous data. A corrected Hedges' *g* was calculated by dividing the difference between group means by the pooled and weighted standard deviation (*SD*) of the groups. Specifically, Hedges' *g* corrects for a bias (overestimation) that occurs when the uncorrected standardised mean difference effect size is used on small samples. We computed a 95% confidence interval (CI) for each combined effect size to test for statistical significance; if the CI did not include zero, we rejected the null hypothesis that there is no difference between the group means.

##### Dichotomous data

We computed Mantel–Haenszel odds ratios (ORs) for the dichotomous outcome variables. Based on the assumption of proportional odds, ORs can be compared between variables with different distributions, including very rare and more frequent occurrences. Specifically, the odds of an event (e.g., children's reunification with their family) were calculated for each sample by dividing the number of children reunified, by the number of children who were not reunified with their family. We then calculated an OR by dividing the odds of reunificiation for the FGDM group by the odds of the non‐FGDM group of children. In addition, we calculated and reported 95% CIs for the ORs reported.

#### 4.3.5 Unit of analysis issues

The unit of analysis for this review was children. There were no unit of analysis issues identified for the included studies.

#### 4.3.6 Dealing with missing data

Although studies with incomplete outcome data (e.g., missing means, *SD*s, sample sizes) were included in the review, they were excluded from the meta‐analyses unless the review authors could calculate an effect size from the available information. When outcome data were missing from an article or report, we made reasonable attempts to retrieve these data from the original researchers. Evidence of attrition of study participants or data is described in the quality assessment of primary studies, reported in “Assessment of risk of bias in included studies” section.

#### 4.3.7 Assessment of heterogeneity

We assessed the consistency of results using the *I* ^2^ statistic (Higgins, 2002, 2003). Evidence of heterogeneity (*p* value from test of heterogeneity < 0.1 coupled with an *I* ^2^ value of 25% or greater) for any of the outcomes synthesised, is highlighted in the accompanying narrative to that outcome reporting.

#### 4.3.8 Assessment of reporting biases

Reporting bias was counteracted to some extent by deploying a highly sensitive systematic search of bibliographic databases, and supplementing this with additional searching of grey literature sources, reference list searching, expert consultation and hand searching. Unpublished data from two separate studies were located through author correspondence, and are included in the review. Primary studies were reviewed for references to a study protocol which could be obtained to check for outcome measures being dropped, or added; just one study report referenced a protocol. Primary study authors' choice of outcomes to study and report were appraised. Only four reported on the continued maltreatment of children. The implications of this are discussed in Selective reporting (reporting bias). The use of a funnel plot, to help identify potential reporting bias in primary studies, was not possible given the small number of study findings synthesised under each outcome heading.

#### 4.3.9 Data synthesis

Meta‐analyses were conducted using RevMan 5. None of the primary studies reported on comparisons between FGDM versions, so all syntheses were completed on the absolute effect of FGDM versus no FGDM. Two studies reported findings from samples separated geographically (from separate child protection agencies, or different territories of the same agency) these data were synthesised as separate studies, because there was a degree of heterogeneity between them.

ORs were used to represent binary outcome data. Continuous data were converted into SMDs. All outcomes were presented with 95% CIs. Hedges’ *g* was used to correct for small sample bias. Where findings for a particular outcome were reported by some studies with continuous data, and with dichotomous data in other studies, the Campbell Collaboration online conversion (Wilson, 2018) calculator was used to convert studies to the majority format.

We assumed there would be unexplained sources of heterogeneity across studies; hence we used a random effects model of meta‐analysis. Results for randomised experiments and quasi‐experimental designs were reported separately. Meta‐analysis was not possible for several of the outcomes reviewed as they were only reported by one or two primary studies. A narrative review is provided for these. Given the small number of studies overall, and the level of heterogeneity between them we did not perceive any opportunities for moderator, sensitivity or outlier analysis. The syntheses completed showed moderate‐to‐high levels of heterogeneity between studies for all outcomes. We deemed the presentation of an overall effect size to be inappropriate for some of the outcomes: when the number of studies synthesised was small, and findings were highly heterogeneous.

#### 4.3.10 Subgroup analysis and investigation of heterogeneity

There were no opportunities to complete a subgroup analysis according to method, FGDM version, population or follow‐up periods. Where studies included findings from interval measures of an outcome, measures taken at the longest time‐period from the intervention were used.

#### 4.3.11 Sensitivity analysis

Due to the small number of primary studies, and limited meta‐analyses completed, there was no opportunity for a sensitivity analysis.

## 5 RESULTS

### 5.1 Description of studies

Table 1 provides an overview of primary study characteristics. The included studies are described in terms of the setting, participants, interventions and outcome measures.

**Table 1:** Summary of primary study characteristics

First author (year)1	Study design	Population	FGDM version	Control condition	*N*	Outcomes	Region	Publication status
Baumann (2005)/Sheets (2009)	Nonrandomised controlled comparison	Children removed from home due to abuse and neglect	Not specified	Permanency Planning Team meeting (which parents can attend)	1,110 Parents and relatives	Parent's and relative's satisfaction with the case planning process	Texas, United States	Published journal article
					83 Relatives or foster parents			
						Relative's or foster parent's views on child well‐being		
					4,066			
						Length of stay in care, and type of permanent placement		
					children			
Berzin (2008)	Randomised controlled trial	Children at risk of further maltreatment and removal	Family unity and family group conferencing	Traditional child protection services without family team meetings	110 children	Service user satisfaction (relatives, parents and children); placement stability; safety‐related outcomes; permanency‐related outcomes	California, United States	Published journal article
Cunning (2006)	Historically controlled comparison (parallel groups)	Children referred to child protection services	Family Group Conferencing	Traditional child protection services	254	Reunification	Toronto, Canada	Published report
						Continued maltreatment		
						Placement stability		
Crampton (2007)	Historically controlled comparison (parallel groups)	Non‐White children who have had a substantiated CPS case, out‐of‐home placement and no sexual abuse	Family Group Decision‐Making	Traditional child protection services	257	Substantiated re‐referrals	Michigan, United States	Published journal article
						Placement stability		
						Reunification		
Dijkstra (2018)	Randomised Controlled Trial	All families referred to child protection services	Family Group Conferencing	Intensive Family Case Management	328	Continued maltreatment	Netherlands	Published journal article
						Expedition of case processing		
						Service user satisfaction		
						Engagement with support services		
Edwards (2006)/Pennell (2010)	Historically controlled comparison (parallel groups)	Children removed from home due to abuse and neglect	Family Team Meetings	Traditional child protection services without family team meetings	789 children	Permanency‐related outcomes, length of foster care, and continued maltreatment	Washington, United States	Agency report and published in a journal article
Hollinshead (2017)/Corwin (2019)	Randomised controlled trial	Child welfare involved families receiving in‐home services	Family group conferencing (Ohana model is referenced)	Traditional child protection services	503 families	Substantiated re‐referrals	Colorado, United States	Two published journal articles
						Maintenance of in‐home care		
						Case‐workers' perception of family's social support		
McRae	Historically controlled comparison (parallel groups)	Children referred to child protection services	Family group conferencing (no further detail is provided)	Traditional child protection services	4,129 children	Referral to and engagement with services	United States (36 states)	Published journal article
Pennell (2000)	Nonrandomised controlled comparison	Children at risk in families with domestic violence	Not specified. Authors refer to both FGDM and FGC (Family Group Conferencing).	Traditional child protection services	63 families (it is unclear how many siblings were in each family)	Permanency outcomes; child safety; child neglect; women abuse	Newfoundland and Labrador, Canada	Published journal article
Sundell (2004)	Nonrandomised controlled comparison	Children involved in child protection services	Authors describe the key principles of FGDM, but do not offer details of the version of FGDM employed	Traditional child protection services	239 children	On‐going service engagement, rate of placement in foster care, type of placement, and case closure	Sweden	Published journal article
Titcomb (2005)	Historically controlled comparison (parallel groups)	Children referred to child protection services	Authors describe the key principles of FGDM, but do not offer details of the version of FGDM employed	Traditional child protection services	540 children	Substantiated reports of abuse within 12 months	Arizona, United States	Published journal article
Walker (2005)	Historically controlled comparison (parallel groups)	Children who are the subject of voluntary foster custody	The Ohana model of Family Group Conferencing	Traditional child protection services	60 children	Permanency of out‐of‐home placement, consumer satisfaction, cost‐effectiveness of conferencing	Hawaii, United States	Published journal article
Wang (2012)	Historically controlled comparison (parallel groups)	Children who had been placed in care for longer than a 3‐day period	The Ohana model of Family Group Conferencing	Not clearly specified. Presumed to be the traditional child protective service process centred on practitioner decision‐making.	80,690	Number of children in permanent placements at 15 months; number of children reunified with their family or placed with relatives at 18 months	Texas, United States	Published journal article
Weigensberg (2009)	Historically controlled comparison (parallel groups)	Children who had contact with welfare services, but who were be cared for at home at baseline	Authors describe the key principles of FGDM, but do not offer details of the version of FGDM employed	Not clearly specified. Presumed to be the traditional child protective service process centred on practitioner decision‐making	5,001 children	Provision of, and engagement with, parent, child and family services	United States, nationwide	Published journal article
Weisz (2006)	Historically controlled comparison (parallel groups)	Children involved in child welfare services	Expedited Family Group Conferencing	Not clearly specified. Presumed to be the traditional child protective service process centred on practitioner decision‐making	66 children	Number of removals during the evaluation period; number of placements; type of most recent placement	Nebraska, United States	Unpublished report

Abbreviation: FGDM, family group decision‐making.

#### 5.1.1 Results of the search

The main bibliographic database search, completed in July 2016, returned 1,320 records. These records were combined with 41 additional records found through reference list searching, hand searching and correspondence with experts and known study authors. This original bibliographic search was re‐run in August 2019, adding 215 search hits. A total of 1,576 studies were subjected to initial screening, 92 of these were selected for full text screening, and 15 of these (describing 13 studies) were found to meet the inclusion criteria for the review. Two additional studies were identified following the 2019 search, through correspondence with primary study authors. Figure 3 offers an overview of search results using a flow diagram. In all, 18 studies reports were selected, describing 15 studies, offering findings for from 18 study samples.

#### 5.1.2 Included studies

Table 1 describes the 15 studies which matched the review selection criteria. Three studies were reported in two study reports. Baumann (2006) and Sheets et al. (2009), reported findings from the same study. Baumann reported findings on the nature of children's placements, Sheets et al. reported some of these findings, but also additional findings related to service user satisfaction. Both study reports were needed to ensure all available findings were obtained. Edwards, Tinworth, Burford, and Pennell (2006) and Pennell (2010) reported findings from the same study of case records. Pennell reported findings for kinship care, expedition of case processing and family reunification; Edwards reported findings on continued maltreatment. Hollinshead (2017) reported on continued maltreatment and family reunification from an randomised controlled trial (RCT), and Corwin et al. (2019) followed up with a further report from this study on case‐workers' perceptions of social support following intervention.

Only four of the included studies were conducted outside the United States; two in Canada (Cunning & Bartlett, 2006; Pennell & Burford, 2000) and one in Sweden (Sundell & Vinnerljung, 2004) and one in the Netherlands (Dijkstra, 2018). Of the fifteen studies reviewed, just three were RCTs (Berzin, 2006; Dijkstra, 2018; Hollinshead, 2017). The other studies employed quasi‐experimental designs, using parallel cohorts. Four of the quasi‐experimental studies used prospective evaluations of nonrandomly assigned comparison groups (Baumann et al., 2005: Pennell, 2000; Sundell & Vinnerljing, 2004; Walker, 2005) the rest analysed pre‐existing survey data, child protection case files or court data. The longest postintervention follow‐up period was 3 years, used by Sundell and Vinnerljung.

Two study reports Berzin et al. (2006) and Cunning and Bartlett (2006) presented findings for separate geographical areas separately. Cunning et al. also reported findings from a combination of the two regions, under one outcome heading. Each grouping was treated as a separate population in the data synthesis.

#### 5.1.3 Excluded studies

Eighty‐five study reports were excluded during the final, full text, screening. Thirty‐eight studies were excluded because the study design did not meet the minimum standards of methodological rigour outline in the review protocol (Shlonsky et al., 2009) and this was, most commonly, because they had no comparison group. Several studies, presented as evaluations, used qualitative data. Tweny‐four studies were excluded because the intervention was not FGDM. The remaining studies were excluded due to: wrong population (nine); insufficient data (five); data being intractably unavailable (two); wrong outcomes (one); and the study has not been completed (one). A list of excluded studies and reasons for exclusion is presented in Excluded studies.

### 5.2 Risk of bias in included studies

**Figure 1:**
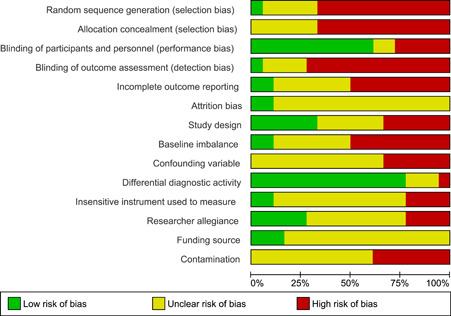
Risk of bias graph: Review authors' judgements about each risk of bias item presented as percentages across all included studies

**Figure 2:**
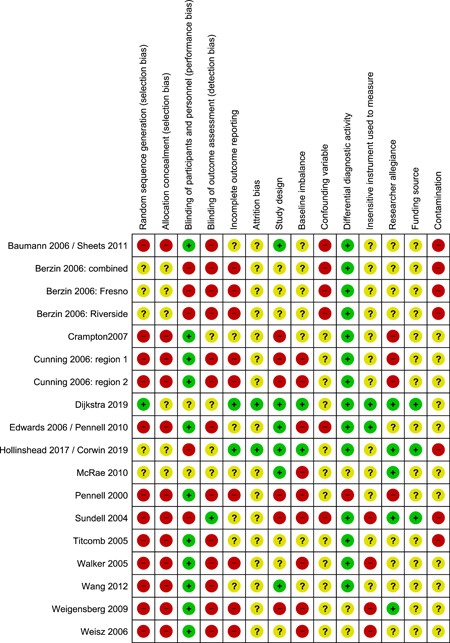
Risk of bias summary: Review authors' judgements about each risk of bias item for each included study

The review authors judged there to be a moderate or high risk of bias in most categories in each of the studies reviewed, see Figure 1 for a summary of judgements on bias across the studies reviewed. Figure 2 provides an insight into the level of potential bias within each study. Appendix A provides the rationale for each of these judgements.

#### 5.2.1 Allocation (selection bias)

Selection bias is comprised of sequence generation and allocation concealment. Studies were rated as high if they failed to provide sufficient information or used comparison groups that represent a population subset. Ten out of the eleven included studies (91%) were rated as having a high risk of selection bias. No studies were rated as low risk, with one study (9%) rated as unclear risk: Berzin (2006) used random assignment, but provided insufficient information to make a judgement on allocation concealment.

#### 5.2.2 Blinding (performance bias and detection bias)

This potential bias is counteracted by the blinding of study participants and personnel, so that they are unaware of their group assignment, and the blinding of outcome assessors. Participants and personnel, in ten of the studies, would have been aware of the type of deployment of FGDM. Only one study report (12%) described the blinding of outcome assessors.

#### 5.2.3 Incomplete outcome data (attrition bias)

Attrition bias refers to the biasing effect of study participants, or study participant data becoming unavailable during the study. This bias can be counteracted by keeping accurate records of participants who drop out of the study, and by using intention‐to‐treat analysis so that drop‐outs do not have a biasing effect on final results. None of the study reports offered information on how families who dropped out of FGDM or comparison treatments were recorded or accounted for in the analysis of findings. For this reason, all of the studies were rated as having an unclear risk of attrition bias.

#### 5.2.4 Selective reporting (reporting bias)

None of the included study reports references a study protocol. We have no way of knowing if some outcome measures were dropped, or added, as the study progressed. Therefore, all of the included studies were judged to have, as a minimum, unclear risk of selective reporting bias. Five studies were judged to have a high risk of incomplete reporting bias, as some findings were clearly missing or only partially described.

#### 5.2.5 Other potential sources of bias

##### Study design bias

If study design choices did not appear to have affected findings for intervention and control groups differentially, study design bias was rated as low. This was the case in three studies (27%). Three studies (36%) were rated as high risk because, variously, study participants self‐selected into study groups or social workers assigned participants to study groups, or the use of FGDM was not adequately confirmed, or study authors referred to qualitative findings as evidence of efficacy. Four studies (36%) were rated as unclear in this category; in these, little or no rational was provided for study design or selection of comparison groups.

##### Baseline imbalance bias

Imbalance at baseline may influence study outcomes and the results of statistical tests. This was rated as high risk in seven of the eleven included studies (64%). The remaining four studies (36%) provided insufficient data, from which to make a judgement, and were rated as unclear.

##### Confounding variable bias

Confounding variables were judged to be a high‐risk factor in four of the included studies (36%). In each case, practitioners were the potentially confounding factor. The remaining seven studies were rated as unclear due to insufficient data being provided. For a low risk of bias in this category, primary study authors would have needed to have offered an assessment of potentially confounding variables, and a description of how they were nullified or dealt with in data analysis.

##### Differential diagnostic activity bias

Studies were rated as high risk in this category, if different measures or collection methods were employed within the intervention and comparison groups. This was the case in one study (Pennell, 2000) whereby data from case files and home visits appear to have been gathered in different ways, for the two groups. It was difficult to discern what was measured over what period in two of the studies. It was not clear how an equivalent date to the FGDM meeting date was established for the comparison group. These two studies were designated unclear. The other eight studies (73%) where there was no evidence of differential diagnostic activity, were assessed as having a low risk of bias.

##### Bias due to the use of insensitive instruments for outcome measurement

Four studies (36%) were rated as high risk of bias for insensitive instruments used to measure outcomes. This included issues regarding the quality and appropriateness of some outcome measures (for example, re‐referrals as a measure of on‐going abuse). Six studies (55%) under‐described their measurement of outcomes so that judgement was difficult in this category. One study was deemed to have a low risk of bias, due to comprehensive reporting of appropriate diagnostic activity.

##### Researcher allegiance bias

Researcher allegiance was rated as high risk of bias in two studies (Cunning & Bartlett, 2006; Pennel & Burford, 2000) the authors argued for the benefits of FGDM within the article, but without supporting references to an appropriate evidence base. Clear information regarding the independence of researchers was provided in only one study (Sundell & Vinnerljung, 2004); the remaining studies (73%) were rated as unclear, due to a lack of information about the independence of researchers from FGDM providers.

##### Funding source bias

A study which is funded by proponents of FGDM, or an agency which has invested in FGDM may be at risk of funding source bias. One of the included studies (Sundell & Vinnerljung, 2004) was conducted by an independent government department charged with the evaluation of social care practice; this study was rated as low risk in this category. The remaining ten studies were rated as having an unclear risk of funding source bias due to insufficient information, or due to funding being provided by the FGDM provider.

##### Contamination bias

Four of the eleven included studies (36%) were given a high‐risk rating of contamination bias. A high‐risk rating was given if the same practitioners delivered both interventions or if the social workers from both FGDM and comparison groups were aware of FGDM provision. For example, social workers, delivering the interventions, were involved in treatment allocation in Sundell and Vinnerljung's (2004) study; Bauman (2006) refers to feedback to staff during implementation, it is possible that learning from the implementation of FGDM was cross‐pollinated to the comparison intervention. The remaining seven studies (64%) were rated as unclear due to a lack of information.

### 5.3 Effects of interventions

#### 5.3.1 Synthesis of results

Meta‐analysis and narrative review were applied to findings from fifteen studies. Sufficient data existed to warrant meta‐analyses under the following outcome groupings: reunification of children with families or maintenance of in‐home care; continued maltreatment; kinship placements; and expedition of case processing and case closure. A narrative review is also offered for findings under the following outcome groupings: placement stability; child well‐being; service‐user satisfaction; and referrals to support services.

#### 5.3.2 Reunification of children with families or maintenance of in‐home care

Ten effect sizes, from nine quasi‐experimental studies, were synthesised to examine effects on the reunification of children with their family, or the effect on maintaining in‐home care; in short, the effect FGDM has on keeping families together. It can be seen from the forest plot (Figure 4) that the dominant finding from the synthesis of these study results is the lack of clarity. There is a high level of heterogeneity between the studies (*I* ^2^ = 92%, see Analysis 1.1); six study findings come with very wide CIs; and CIs for six out of the ten studies span the line of no effect. The overall effect, based on the combination of these studies is small but statistically significant: OR, 1.69 (CI, 1.03, 2.78); test for overall effect significance: *Z* = 2.07 (*p* = .04). Thus, children in the FGDM groups had better odds of being with their family of origin at the end of the study period, their odds were 1.7 times greater.

**Figure 3:**
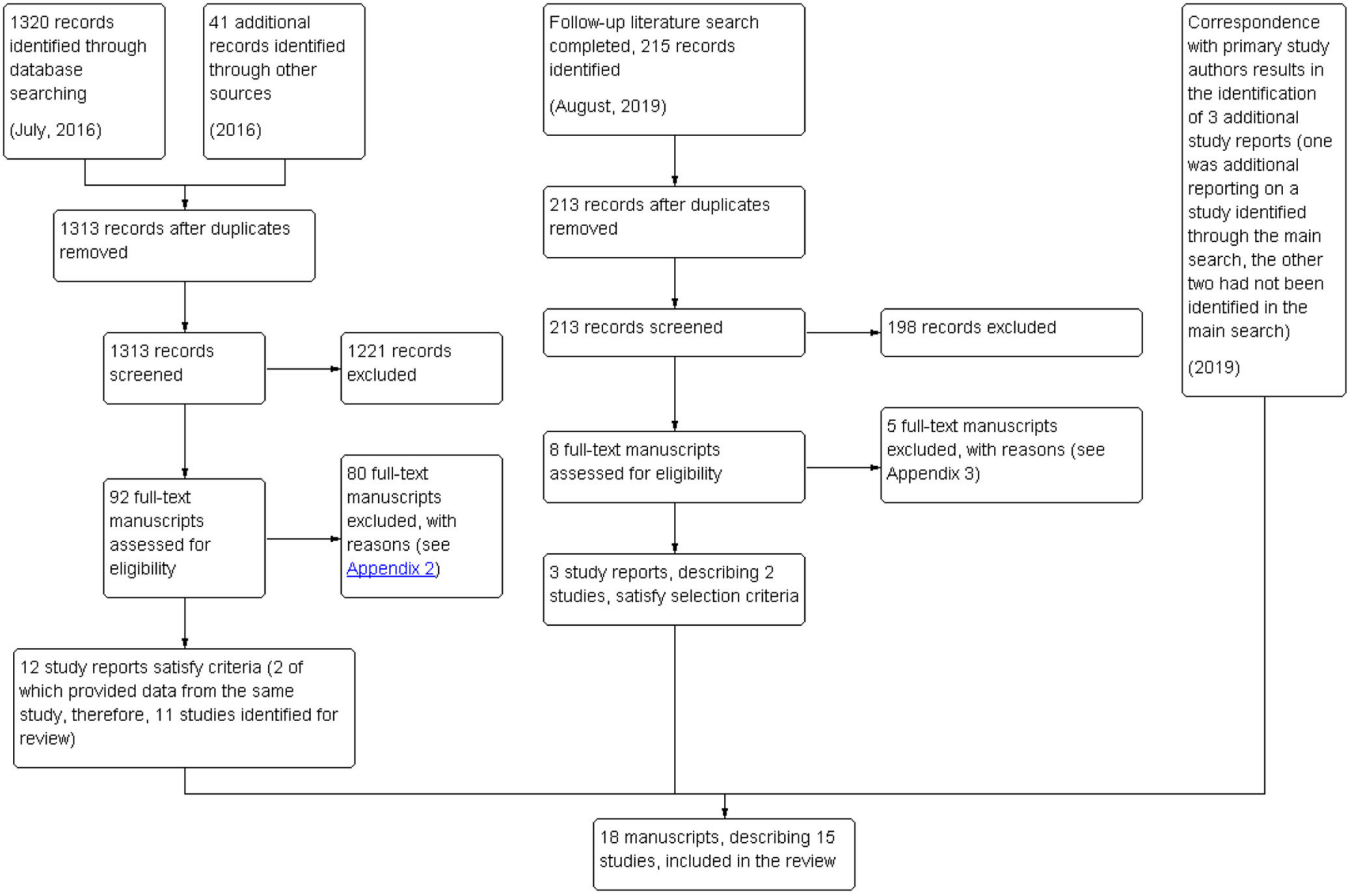
Flow chart of study selection process

**Figure 4:**
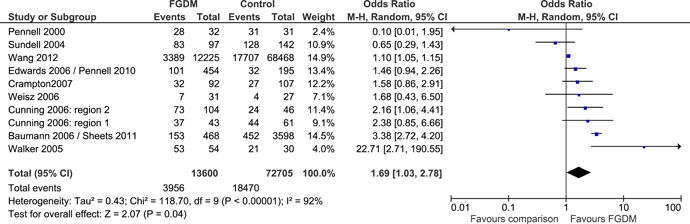
(Analysis 1.1) Forest plot of comparison: 1 Traditional child protection case processing, outcome: 1.1 1.1 Reunification of children with families or maintenance of in‐home care. CI, confidence interval; FGDM, family group decision‐making; OR, odds ratio

Hollinshead's (2017) RCT reported a similar finding to that found in the quasi‐experimental studies. Hollinshead's, intention‐to‐treat analysis, of out‐of‐home placements found a positive result but it was not statistically significant: OR, 0.68 (CI, 0.24, 1.94; *p* > .05).

#### 5.3.3 Continued maltreatment

Meta‐analysis of five quasi‐experimental studies, which reported the number of children who continued to be maltreated, following FGDM or traditional child protection decision‐making procedures, is provided in Analysis 1.2. It can be seen that just one study recorded significantly lower incidents of continued maltreatment (Sundell & Vinnerljung, 2004). While Pennell's (2000) small study (*n *= 63) reported a negative effect for the FGDM group. The overall effect, based on the combination of these studies favoured the FGDM group, but was not statistically significant: OR, 0.73 (CI, 0.48, 1.11); test for overall effect significance: *Z* = 1.48 (*p* = .14).

Berzin et al's (2008) RCT studied reported maltreatment in two separate geographical areas, see Analysis 1.3. Results were similar across both studies. There were significantly fewer reports of continued maltreatment in the control conditions. A large RCT (*N *= 523), conducted by Hollinshead (2017) reported very few re‐referrals for either FGDM or traditional child protection work, and little difference between the two conditions. While Dijkstra, Asscher, Deković, Stams, and Creemers (2019, *N *= 328) reported a nonsignificant positive effect for FGDM. The overall combined effect, reported in RCTs favoured the control group, but was not statistically significant: OR, 1.29 (CI, 0.85, 1.98); test for overall effect significance: *Z* = 1.2 (*p* = .23).

In addition, to the analyses presented in Analysis 1.2 and Analysis 1.3, Walker (2005) and Cunning and Bartlett (2006) both report ratio data relating to continued maltreatment. Walker (2005) reported the average number of emergency placements in a children's shelter. Children from the FGDM group were placed in a children's shelter an average of 0.24 times (*n *= 54; *SD* = 1.3) during the study period, in comparison to an average of 1.0 times (*n *= 30; *SD* = 1.3) for children in the comparison group (these *SD*s were computed using the reported *p* value, according to Cochrane guidance, Higgins & Green, 2009). Cunning et al. found that conferenced children's cases were reopened an average 0.3 times (*n *= 30; *SD* = 0.95) as opposed to an average of 0.38 for non‐conferenced children (*n *= 41; *SD *= 0.76) in one area. In another area, Cunning found this result was reversed: conferenced children's cases were reopened an average 0.43 times (*n *= 67; *SD* = 0.95) as opposed to an average of 0.30 (*n *= 48; *SD *= 0.63) for non‐conferenced children.

An overall effect size, based on the 10 studies referred to here, is not offered because of the lack of conformity in data types, study designs, and because of the level of heterogeneity across the study data synthesised in Analyses 1.2 and 1.3. The data pertaining to FGDM and continued maltreatment could be summarised as inconclusive.

#### 5.3.4 Kinship placements

Five effect sizes, from nonrandomised studies, were synthesised to examine the effect of FGDM on the number of kinship placements. It can be seen from Analysis 1.4 that there was a high level of heterogeneity between the studies (*I* ^2^ = 74%); two study findings had very wide CIs; and CIs for four out of the five studies span the line of no effect. The overall positive effect based on the combination of these studies is negligible: OR, 1.31 (CI, 0.94, 1.82). and primarily a reflection of Wang et al.'s (2012) finding (weighted at 96%).

Walker (2005) also reported findings of a positive effect on kinship placements. Walker reported ratio data, without enough information to compute an OR, for inclusion in Analysis 1.4. In Walker's study, the average number of times children were moved to a kinship placement for the FGDM group was 0.68 (*n *= 54) and for the non‐FGDM group it was 0.95 (*n *= 30).

#### 5.3.5 Expedition of case processing and case closure

Because *SD*s for Weisz, Korpas, and Wingrove (2006) and Sundell & Vinnerljung (2004) were unavailable, and a *SD* for Walker (2005) was approximated using Cochrane guidance (Higgins & Green, 2009) the provision of a study heterogeneity statistic, or overall effect size, was not possible. Table 2 provides an overview of findings from all six (quasi‐experimental) studies on case processing speed or case closure. It can be seen that study findings for this measure are wide‐ranging and inconsistent.

**Table 2:** Number of days to case closure/case processing target

	*n*	*d*	95% CI	95% CI	FGDM cases were processed quicker? (Yes/No)
RCTs					
Berzin (2008)	50	3.56	−1.10	8.22	No
Dijsktra (2019)	328	0.59	0.35	0.83	No
Quasi‐experimental studies					
Weisz (2006)	66	−0.51	0.06	−1.07	Yes
Sundell (2004)	170	0.48	−0.83	−0.12	No
Walker (2005)	84	−8.30	−13.08	−3.52	Yes
Edwards (2006)/Pennell (2010)	649	−0.27	‐0.03	−0.50	Yes

*Note*: Pennell (2010) reported dichotomous data for leaving care at 180 days. These data were converted to ratio data using the Campbell Collaboration online conversion (Wilson, 2018) calculator.

Abbreviations: CI, confidence interval; FGDM, family group decision‐making; RCT, randomised controlled trial.

#### 5.3.6 Placement stability

Table 3 summarises study findings pertaining to placement stability. Berzin's (2008) RCT, reported on the stability of children's placements. Berzin recorded the number of placement moves participant children endured during the study period. The FGDM group were moved an average of 0.75 times (*SD* = 1.17, *n *= 108) while the control group were moved an average of 0.55 times (*SD* = 1.14, *n *= 119). The mean difference in placement moves for the two groups favoured the control group, but not at a statistically significant level: *d *= 0.20 (CI, −0.10, 0.50).

**Table 3:** Placement stability

	*n*	*d*	95% CI	95% CI	FGDM cases had less placement moves? (Yes/No)
RCTs					
Berzin (2018), combined	227	0.20	−0.10	0.50	No
Quasi‐experimental studies					
Crampton (2007)	257	−0.56	−0.05	−1.08	Yes
Cunning (2006), region 2	150	−0.31	−1.58	0.96	Yes
Cunning (2006), region 1	104	−0.37	−1.35	0.61	Yes

*Note*: Crampton (2007) compared the number of FGDM children who had three or more moves with children who had three or placement moves in the non‐FGDM group. Berzin (2018) and Cunning and Bartlett (2006) compared average number of placement moves.

Abbreviations: CI, confidence interval; FGDM, family group decision‐making; RCT, randomised controlled trial.

Three quasi‐experimental studies also reported findings for child placement stability. Crampton's (2007) study found a moderate positive effect for FGDM children: *d* = −0.56 (CI, −0.05, −1.08). Cunning and Bartlett's (2006) study in two separate areas, also found moderate positive effects for FGDM: *d* = −0.31 (CI, −1.58, 0.96); and *d* = 0.37 (CI, −1.35, 0.61).

#### 5.3.7 Service user satisfaction

Walker (2005) compared responses from 30 individuals, from 30 families, 13 of which had been involved in a traditional CPS process, 17 of which had been involved in an FGDM meeting while their case was open: 41% of the FGDM group described the process they were involved in as positive, in comparison to 23% of the non‐FGDM group.

Sheets et al. (2009) compared survey responses from FGDM and non‐FGDM parents and relatives under three headings: satisfaction with family plan; understanding of what was expected of them; and sense of empowerment. Data were collected using an unvalidated 5‐point rating scale and sample sizes ranged from 50 to 636. The actual sample sizes and *SD*s, used in each comparison, were not reported. Parents and relatives who had been involved in the non‐FGDM group reported statistically significant (*p *< .001) higher levels of satisfaction in all three areas.

Dijkstra (2018) found that perceptions of empowerment, 12 months after a care plan had been agreed, did not differ between FGDM and non‐FGDM groups of parents.

#### 5.3.8 Engagement with support services

Dijsktra's (2019) RCT found that FGDM had a small positive effect on families' engagement with services, 12 months after a care plan had been agreed: FGDM families were involved with an average ot 2.24 services in comparison to 1.78 services for non‐FGDM families (*d* = 0.22; CI, −0.01, 0.46). Weigensberg et al. (2009) utilised 36 months of nationally‐representative data from the U.S. National Survey of Child and Adolescent Well‐Being (NSCAW Research Group, 2002) to evaluate the impact of FGDM meetings on children's and families' involvement in intervention and support. They compared data on 325 children who experienced FGDM and a propensity score matched (Rosenbaum & Rubin, 1983) comparison group of 325 non‐FGDM children. A higher percentage of children who experienced FGDM meetings were connected with services initially. After 36 months, however, receipt of child and family services was not statistically different between children who experienced FGDM meetings and those who did not.

#### 5.3.9 Social support

Dijkstra et al. (2019) RCT found that parents' perception of their social support 12 months after a care plan had been agreed was higher for FGDM families. Parents in the FGDM group scored their level of support at 3.43 (*SD* = 0.47) on a 4‐point scale; in comparison to 3.40 (*SD* = 0.57) for non‐FGDM families.

Hollinshead's (2017) RCT measured case‐workers' perception of familes' social support using a 5‐point scale. The average rating from case‐workers, working with FGDM families', was 2.7. Higher than the average rating of 2.1 from workers working with non‐FGDM families (*SD* was not provided; *N *= 503).

## 6 DISCUSSION

### 6.1 Summary of main results

A high level of heterogeneity between primary studies, and a high risk of bias across primary studies, are the foremost findings of this review. Any discussion of overall effect sizes is overshadowed by these two key review findings.

The primary outcomes of interest, as outlined in the review protocol (Shlonsky et al, 2009) were FGDM effects on child maltreatment, family permanence and placement stability. The synthesis of study findings provided here, in relation to these outcomes, is inconclusive. While a meta‐analysis of ten quasi‐experimental study findings provides a small overall effect size on the reunification of children with their families, we suggest that this should not be held as evidence of FGDM efficacy, for several reasons. The wide‐ranging CIs within some of the studies, and the wide range of findings across studies, suggest limited reliability for these findings. When considered alongside a high risk of bias across the studies, these shortfalls detract greatly from the importance of an overall small effect size.

Evidence of the effect FGDM has on continued maltreatment is also inconclusive. Four out of five nonrandomised studies found that FGDM reduced the likelihood of further maltreatment, but a meta‐analysis of these was not statistically significant. Three RCTs (including four study samples) were also pooled using meta‐analysis. FGDM children were maltreated more often in two study samples, and less often in the other two. The overall effect was small and not statistically significant. Similarly, there is no clear direction in relation to placement stability. While FGDM was found to have a moderately positive effect on placement stability in two quasi‐experimental studies, a negative effective is reported by a similar sized RCT.

Evidence of the effect FGDM has on kinship placements is heterogeneous across the five nonrandomised studies synthesised. The meta‐analysis favours FGDM but is not statistically significant. Similarly, there is little convergence between four studies reporting on the expedition of case processing and case closures.

Evidence of the effect FGDM has on family group type permanency goals, service user satisfaction, child well‐being and on engagement with support services is also inconclusive. Just one or two studies reported on each of these outcomes, and overall effect sizes were not calculable.

### 6.2 Overall completeness and applicability of evidence

The evidence available does not form a complete picture. It is predominantly U.S. based. It offers scant information relating to the integrity of FDGM deployment. There are significant risks of bias in most of the studies reviewed. In addition, to these issues of external validity, the synthesis of primary study findings has not suggested an overall effect, either positive or negative. These are insights which can inform child protection policy. More specifically, this reviews suggest that FGDM should be rigorously evaluated (including the evaluation of treatment integrity) where ever it is used.

### 6.3 Quality of the evidence

Risk of bias in included studies provides a detailed description, and summary, of primary study bias judgements. Study rigour was low across this body of evidence. Very few of the mechanisms of rigour encouraged by guidance for the conduct of intervention evaluations (e.g., Rychetnik, Frommer, Hawe, & Shiell, 2002; Viswanathan et al., 2017; White & Sabarwal, 2014) were described by primary study authors. Study participant selection bias, baseline imbalance bias and reporting bias were the most significant detractors from the internal validity of the studies reviewed; these were rated as high in a large majority of the studies. One study by Sundell and Vinnerljung (2004) was judged to have a high risk of bias in eight out of the fourteen categories assessed. The average number of high bias ratings per study was 5.9. We would suggest that the range and extent of potential bias in this body of evidence is cause for caution in judging the efficacy or harm of FGDM interventions.

Also, in relation to the quality of evidence, we should acknowledge that the body of evidence is small: in terms of eligible studies; and due to the limited overlaps in outcome measures used across the dataset. Only one of the outcomes of interest, reunification, was reported by a majority of the nine studies. From another perspective, the body of evidence reviewed here is substantial, it includes data from over 93,000 study participants. This compares favourably to the majority of reviews published by the Cochrane and Campbell collaborations. However, these participant data are predominantly gathered from large retrospective cohort studies, using secondary data.

### 6.4 Potential biases in the review process

We could not identify any potential biases in the current review process.

### 6.5 Agreements and disagreements with other studies or reviews

The current review findings disagree with narrative reviews by Crampton (2006), Frost et al. (2014) and Merkel‐Holguin et al. (2003) who put a positive slant on evaluations reviewed, with little critique of primary study rigour. The current review agrees with Havnen & Christiansen (2014) and Dijkstra et al. (2016) who highlight a low level of rigour across the evidence base, and inconclusive findings.

## 7 AUTHORS' CONCLUSIONS

### 7.1 Implications for practice

The methodological rigour across this body of evidence must be described as low. The risk of bias among primary studies is high. The range of outcomes reported offers limited opportunity for meta‐analyses. The small meta‐analyses, completed here, brought together quite heterogeneous findings. In these circumstances, the current review authors would emphasise that there is insufficient evidence to support a judgement on the efficacy of FGDM, for the prevention of abuse and neglect of children. Tukey (1986, p. 74) stated that “the combination of some data, and an aching desire for an answer, does not ensure that a reasonable answer can be extracted from a given body of data”. While we have been able to combine data from separate studies, on a number of outcomes of interest, we believe that it would be misleading to suggest that these meta‐analyses provide answers to questions of FGDM efficacy.

Considering how the findings of this review contrast to the American Humane Society's (Merkel‐Holguin et al., 2003) narrative review, leads us to the question of why the prevailing sentiment on FGDM is so positive. We would suggest that Merkel et al.'s conclusions reflect common sentiments among practitioners and stakeholders who have been involved in implementing FGDM projects: that FGDM is based on sound theoretical underpinnings, humanistic (Horwitz & Marshall, 2015) and systems theory (Holland & Rivett, 2006); that FGDM aligns with social work values and aspirations such as partnership in practice (Lohrbach, 2003) and strengths‐based intervention (Connolly, 2005); and that FGDM is an explicit recognition of family's rights (Edwards & Sagatun‐Edwards, 2007). The current review authors concur that the theoretical underpinning for FGDM is logical. Like the authors referenced here, and many others besides, we can see how FGDM has emerged as a logical step in the development of child protection practice. However, the findings of this review give us pause, to consider, why do we believe outcomes are improved with FGDM?

We would point towards commentary that highlights how little we know about what works in child protection work: “It is a sad fact that scientific knowledge of truly effective interventions in child protection is relatively sparse” (Sundell & Vinnerljung, 2004, p. 282). In this situation, it is not inconceivable that policy makers and practitioners have accepted the best evidence they have to hand. Policy makers charged with the allocation of resources for child protection should therefore consider the commissioning of rigorous evaluations of FGDM and non‐FGDM methods of decision‐making. The prevailing sentiment, that FGDM is preferable to other approaches to decision‐making, should be set aside pending appropriate evaluation.

Drawing on commentary of primary study authors, we can suggest several potential reasons for the equivocal performance of FGDM models in comparison to traditional practitioner‐led decision‐making models. These insights may inform the development of practice and its evaluation in this field.

First, let us consider that the success of the decisions, and action plans, put forward by families may be dependent upon the resources available to support these decisions and action plans (as suggested by Sundell & Vinnerljung, 2004). While we might assume that a lack of services, such as counselling, respite or specialist assessments will affect FGDM children and non‐FGDM children equally, we could also conceive that a family which has successfully used the FGDM model are proffered more autonomy to make their own plan happen. Reduced practitioner focus on FGDM plan implementation would have a negative and confounding effect on FGDM outcomes.

Second, there is the possibility that the support offered by family, extended family and the community during the FGDM process is not fully realised. Sundell and Vinnerljing (2004) question if FGDM can make a lasting difference when child welfare authorities attempt to mobilise, informal, networks of children at risk? C. S. M. Lupton (2003); Marsh and Crow (1998); Pennell and Burford (2000); Shore, Wirth, Cahn, Yancey, and Gunderson (2002); and Sundell and Haeggman (1999) all report some level of qualitative feedback, or survey data, from FGDM participants that promised family supports which did not materialise in the manner expected.

Third there is a question mark over the readiness of social work departments, and individual social workers, to embrace FGDM's deference to family decisions, and the family plan (Frost et al., 2014). For example, private family time is not always facilitated, for example in Riverside County, California (see Berzin, 2006). Vesneski (2009) report fear of speaking up, Adams and Chandler (2004) report how family plans are often rejected or changed by child protection workers.

A key question for policy makers, and practitioners, is whether or not they can incorporate a model like FGDM into their practice when their practice is, by necessity, inherently risk averse (Morris & Connolly, 2012)? Child protection policy is heavily influenced by past mistakes, and subsequent serious case reviews. At this point, in any given jurisdiction, there are a variety of reports on previous maltreatment cases which show how more action should have been taken to protect children at risk. In such a risk averse environment is there a role for a model of decision‐making, and planning, which places practitioners on the periphery; and asks the family, within which abuse or neglect has been perpetrated, to divine the best way forward? We would argue that there is.

While we have called for more rigorous evaluation of FGDM, and a process of FGDM development in response to more rigorous evaluation, we would hope that the findings reported here do not contribute to a side‐lining of FGDM. Service users prefer FGDM (Sheets et al., 2009; Walker, 2005). Practitioners who engage with FGDM are also positive about it as they have found it reduces conflict between practitioners and families (Wick, 2014). “A child protection system that uses these models (FGDM and similar) and, where possible, draws upon family strengths as a part of a spectrum of responses to different situations that arise during the life of a child's case, will serve the child, the family, and the community in a more nuanced and effective way” (Edwards & Sagatun‐Edwards, 2007, p. 20). In concurrence with Edwards and Sagatun‐Edwards going forward, we believe it is likely that policy makers will adopt criteria for the allocation of FGDM service to appropriate child protection cases. While it is unlikely to be appropriate as a blanket response to all cases of neglect and maltreatment, in any jurisdiction, its potential as a strengths‐based family intervention may be fully realised through further development and evaluation.

Finally, let us consider the possibility that FGDM cannot have a large impact on outcomes for children. Not because there is anything in particular wrong with it, but because improving outcomes for children at risk of abuse and neglect is very difficult to achieve. Child abuse and neglect correlates strongly with poverty, deprivation, and displacement (Aber, Bennett, Conley, & Li, 1997; Myers, 2002). Child abuse and neglect is at least partly subject to intergenerational transmission (Lo, Chan, & Ip, 2017). Any intervention which provides us with even marginally better outcomes, in the face of society‐wide seemingly intractable challenges such as these, is to be embraced: “FGDM may not be a strong enough intervention to effectively improve child welfare outcomes or may be just one step in improving these larger outcomes” (Berzin, 2006, p. 1456). Policy makers who are looking for step change in outcomes, for maltreated children, are more likely to find satisfaction in responding to the persistent message from practitioners and researchers in this field: who call for manageable case‐loads and adequate long‐term support services for the families they work with. Berzin (2006, p.1456) makes the point thus: “The lack of effects on outcomes may also be attributed to systemic poor service delivery, for which FGDM would have had too limited an influence”.

### 7.2 Implications for research

Sundell and Vinnerljung (2004) explain that due to both political and practical reasons, an RCT was not an option in their study. Frost et al. (2012) argue that RCTs oversimplify the relationships between cause and effect. Frost et al. argue that concepts such as child abuse are socially constructed and interventions such as FGDM are difficult to delineate, and cites these as possible barriers to the application of scientific method in this area. We would suggest that child abuse is no more socially constructed than concepts such as anxiety or depression. Interventions for anxiety and depression have been evaluated and systematically reviewed for decades. We would argue that the same can be done for child abuse interventions, such as FGDM. In addition, cognitive behavioural therapy is widely accepted as an effective response to anxiety and depression. Cognitive behavioural therapy is arguably more complex and vulnerable to confounding variables than FGDM, yet, it has been subjected to countless RCTs and extensively improved and developed through this research.

“The results of randomised clinical trials move the field forward” (Berliner, 2005, p. 104). Berliner describes her evaluation of Trauma Focused—Cognitive Behavioural Therapy (TF‐CBT) with maltreated children. Berliner reports a rigorous evaluation built on learning from previous studies, amounting to a persistent line of enquiry which proves the value of TF‐CBT against criteria for scientific validity. Berliner highlights that “all of this turns out to work just fine even in the messy world of child maltreatment” (p. 104). In short, although we argue that rigorous RCTs of FGDM are possible, the real question is how can we harness the resources necessary to complete them?.

In addition to the resources needed to complete rigorous evaluation, the additional resources required to facilitate FGDM must also be found. The small numbers of families using FGDM in most countries is a key barrier to rigorous evaluations. Four of the studies included in this review had comparatively small sample sizes (less than 50 participants), those with large sample sizes were retrospective studies of pre‐existing datasets. One problem which is reported in commentaries on FGDM implementation is the difficulty in encouraging social workers to use FGDM, when it is made available. Crampton (2006) describes two abandoned attempts, one on each side of the Atlantic, to complete RCTs due to a lack of study participants. Policy makers should work with researchers to ensure projected FGDM take‐up materialises when the model is introduced by child protection departments. Frost et al. (2012) describe the extensive support required to initiate wholesale uptake of FGDM by child protection workers. Measures described include making access to FGDM training for practitioners competitive rather than compulsory; establishing goals for FGDM take‐up and publishing departmental progress against these goals; providing flexi‐time and over‐time to social workers facilitating conferences; and not allowing FGDM plans agreed by social workers to be over‐ruled by their managers.

If researchers can secure appropriate sample sizes for their studies, then this would help to mitigate against the range of potential biases found in the studies reviewed here. Some of these biases can easily be avoided. For example, the blinding of outcome assessment is important in this field because the outcomes in question are often open to interpretation. Qualitative judgements are made on whether or not a subsequent incident of abuse is substantiated or not. Whether data for the assessment of outcomes is drawn from case files, study participant surveys, or practitioner surveys, it is likely that it will need to be anonymised and passed on to a second researcher who has no way of knowing to which trial arm any particular datum belongs (see Schulz & Grimes 2002, for illustrations of blinding in the evaluation of more complex interventions). In addition, data analysts should also be unaware of trial arms’ true identities (Karanicolas, Farrokhyar, & Bhandari, 2010).

In addition to the biases which are explicitly the responsibility of the researcher, the current review identified several potential biases related to the delivery of FGDM and comparison services. The existence of multiple versions of FGDM expands the volume of research needed to evaluate FGDM, and necessitates the use of subgroup analysis in the systematic review of FGDM. It is not a barrier to the evaluation of FGDM as long as researchers are thorough in their description of the FGDM model used, and the fidelity of its implementation is monitored and reported. To avoid charges of treatment fidelity bias, therefore, researchers should provide adequate descriptions of the FGDM model deployed, and the monitoring or its delivery (Robb, Burns, Docherty, & Haase, 2011; Robb, 2011 provide in‐depth guidance on treatment fidelity monitoring). A credible system of monitoring service delivery will also prevent treatment fidelity issues, and help identify potential confounding factors.

The independent monitoring of service delivery will also counteract the confounding effect of cross‐pollination between trial arms. Readers with experience of working in child protection services will be aware that an initiative designed to improve practice, launched in one part of the service, is likely to be discussed and drawn upon throughout the service, even though it has not been fully implemented across the service. Using separate staff for separate trial arms is therefore important, as is the monitoring of FGDM and comparison service delivery.

Finally, in relation to biases incurred during the delivery of FGDM and comparison services, selection bias was arguably the most problematic aspect of the body of evidence summarised in this review. Evidence from a number of studies on FGDM implementation suggest that certain types of cases are referred to the FGDM service, while other types of cases are unlikely to be referred (Wick, 2014). Walker (2005) describes how this might be avoided. In simple terms, families involved in cases which satisfy a criteria for FGDM referral could be asked whether they would be willing to participate in a conference. They would be told that there is a 50% chance of being selected to participate, and a 50% chance of being part of a comparison group. With this prior consent, families could then be assigned to FGDM or the alternative, on a random basis.

Following the conduct of this review, we believe we are in a position to add to the discussion on two potentially powerful confounding variables that various commentators have highlighted. Namely, the confounding effect of family's action plans not being implemented, and the confounding effect of increased reporting of abuse, due to increased involvement of extended family.

The degree to which family's’ action plans are implemented, is vitally important. Berzin (2006) cited family follow‐through on agreed actions as instrumental in the success of FGDM. Berzin et al. (2007) extended the monitoring of FGDM to a follow‐up period which provided data on how plans agreed during FGDM meetings were implemented. Policy makers, practitioners and researchers need to be clear about the level of support families in both arms of a trial are to be given, subsequent to the decision‐making process. The delivery of this support should be monitored. Under‐supported families in either arm of the trial will clearly have a confounding effect on outcomes for children at risk. Frost et al. (2012) suggest that longer study time‐frames of properly supported families are needed.

Researchers should also be aware of the potentially confounding effect of increased reporting of abuse in the FGDM arm, due to deeper involvement of extended family in the cases. Sundell and Vinnerljung (2004) found that significantly more FGDM children were re‐referred to protection services during a 3‐year follow‐up period, than non‐FGDM children. Sundell and Vinnerljing acknowledged the possibility that FGDM might have led to increases in referrals, given that family members would be more aware and more likely to report abuse, but clarified that few children in their study were re‐referred by extended family members. The point remains, however, re‐referrals may be an indication of more diligent monitoring of child welfare, as opposed to a robust indicator of the success of any given intervention. Weinberger, Oddone Eugene, and Henderson William (1996) describe a similar counter‐intuitive effect of intervention in relation to veterans access to primary care. Weinberger et al. found that increased access to primary care increased the rate of hospitalisation. Their study also found that participants who experienced increased access to primary care were more satisfied. If we align this insight with the increased reporting of abuse following FGDM intervention, we are minded to question what the most appropriate outcome of interest for children at risk is? Higher rates of re‐referral for children who have experience FGDM may in fact be indicative of better welfare monitoring. In this scenario, the primary outcome of interest then becomes measures of well‐being, and quality of life indicators.

In summary, of the authors’ conclusions to this review, the current review neither proves nor disproves the efficacy of FGDM. Primary study findings are largely equivocal, and the evidence base is of low quality. Going forward, there is much to learn from the analysis of potential bias presented in this review. We argue that RCTs can be completed in this area. A host of study design features, such as those discussed here, can be adopted to make future evaluations of FGDM highly rigorous. Previous reviewers (Crampton, 2007; Frost et al., 2014; Havnen & Christiansen, 2014; C. Lupton & Nixon, 1999; Merkel‐Holguin et al., 2003; van der Put, Assink, Gubbels, & van Solinge, 2017; Wick, 2014) began the work of unpicking what might make the difference in FGDM implementation. We would encourage future researchers to engage with these resources, avoid the potential biases incurred by the studies reviewed here, and to conduct rigorous evaluations of FGDM in accordance with accepted best practice in scientific enquiry.

## AUTHOR CONTRIBUTIONS

### Content

Tony McGinn and Aron Shlonsky

### Systematic review methods

Manuscript screening: Tony McGinn, Paul Best, and Jason Wilson

Data extraction from included studies: Tony McGinn, Mphatso Kamndaya and Admire Chereni

Assessment of risk of bias: Tony McGinn and Paul Best

### Statistical analysis

Tony McGinn and Aron Shlonsky

### Information retrieval

Tania Celeste and Frances Morrissey, Scholarly Information (Brownless Biomedical Library), University of Melbourne; Tony McGinn, Jason Wilson, Admire Chereni and Paul Best.

## CONFLICT OF INTERESTS

The authors declare that there are no conflict of interests.

## DIFFERENCES BETWEEN PROTOCOL AND REVIEW

There were three differences between the protocol (Shlonsky et al., 2009) a priori guidance for the conduct of this review, as follows.

### Assessment of bias categories

The protocol outlined five categories of bias. The assessment of research bias has advanced significantly, since the protocol was written in 2009, and the five categories of bias described in the protocol were sub‐divided and additional categories were added. Fifteen categories of bias were used in the review.

### Intervention and comparator definition

Policy and practice guidance in most developed nations now acknowledges the importance of in‐depth engagement with children's families and extended families where possible. In the decades since FGC emerged there has been some convergence between the development of traditional child protection decision‐making and the development of FGDM models. The review authors found it necessary to clarify the factors which separate FGDM and decision‐making models which are more practitioner‐driven. Studies included in this review compared: FGDM models which had independent, FGDM‐trained chairs, for meetings with private family time and a prioritisation of family‐proposed plans; with child protection work that did not involve these components.

### Outcomes reviewed

We extended the range of secondary outcomes of interest. We reviewed data pertaining to families’ engagement with services, and families’ perceptions of support. These data were available in four primary study reports. The omission of these outcomes from the review protocol was deemed an oversight. These data made no difference to the overall conclusion of the review.

## PUBLISHED NOTES

### Characteristics of included studies

Baumann (2006)/Sheets 2011

**Table:** 

Methods	Retrospective parallel cohort study
Participants	Children who have been removed from their home for abuse and neglect
Interventions	Family Group Decision Making
Outcomes	Parent's satisfaction (sense of empowerment facilitated by the process)
	Relative's satisfaction (sense of empowerment facilitated by the process)
	Parent's satisfaction (understanding of what was expected of them)
	Relative's satisfaction (understanding of what was expected)
	Parent's satisfaction (with the family plan)
	Relative's satisfaction (with the family plan)
	Number of children exiting care
	Number of children reunified with their family
	Number of children placed with relatives
Notes	

Risk of bias table

**Table:** 

Bias	Authors' judgement	Support for judgement
Random sequence generation (selection bias)	High risk	This was a retrospective parallel cohort design
Allocation concealment (selection bias)	High risk	Not achievable with this study method
Blinding of participants and personnel (performance bias)	Low risk	Study participants and practitioners were unlikely to have been aware of the study during intervention
Blinding of outcome assessment (detection bias)	High risk	Study participants and practitioners were unlikely to have been aware of the study during intervention
Incomplete outcome reporting	Unclear risk	A study protocol, in which data collection and analysis would have been described, is not referenced
Attrition bias	Unclear risk	Insufficient information
Study design	Low risk	There is no evidence that the study designed advantaged the FGDM or comparison group
Baseline imbalance	Unclear risk	Insufficient information
Confounding variable	High risk	Social workers deemed some cases to be unsuitable for FGDM
Differential diagnostic activity	Low risk	Diagnostic activity appeared to be similar for both FGDM and the comparison group
Insensitive instrument used to measure	Unclear risk	The data collection point was not precise: five to seventeen months after the child was placed in care
Researcher allegiance	Unclear risk	Possible examples of a lapse in objectivity, in the reporting of study findings, can be found in both the Sheets and Baumann study reports. Authors do offer some appraisal of study limitations
Funding source	Unclear risk	Insufficient information
Contamination	High risk	Authors refer to feedback to staff during implementation. It is likely that learning from the implementation of FGDM cross‐pollinated to the comparison intervention, Permanency Planning Team meetings

Berzin (2006): combined

**Table:** 

Methods	RCT
Participants	Children ages birth to 18 years who were assessed as being at moderate to high risk for further maltreatment and whose families were eligible for voluntary in‐home services
Interventions	Family Team Meetings (blends family unity and family group conference models)
Outcomes	Number of substantiated reports of maltreatment
	Impact on rate of removal from the home
	Placement stability
	Case closure for a positive reason
	Average time to permanency (case closure) for those case which were closed
Notes	

Risk of bias table

**Table:** 

Bias	Authors' judgement	Support for judgement
Random sequence generation (selection bias)	Unclear risk	Authors describe the study as a randomised controlled trial. A ratio of three treatment allocations to two control allocations is described. No further details of sequence generation are provided
Allocation concealment (selection bias)	Unclear risk	Insufficient information
Blinding of participants and personnel (performance bias)	High risk	Both study participants and practitioners were aware that they were involved in either the treatment or control arm of the experiment
Blinding of outcome assessment (detection bias)	High risk	Authors make no reference to the blinding of outcome assessors
Incomplete outcome reporting	High risk	A study protocol is not referenced. The narrative summary provided omits key data such as effect sizes, and variance measures. Data were only available from one of the two counties involved in the trial, for measures of placement permanency
Attrition bias	Unclear risk	Insufficient information
Study design	Unclear risk	The use of “sibling data” to inflate sample sizes, followed by the use of general estimating equations to counteract clustering effects, and the use of a fixed effects model, could be argued to be inappropriate analyses
Baseline imbalance	Unclear risk	A significantly higher number of children in the treatment group were female Descriptive statistics for the samples at baseline are not provided
Confounding variable	High risk	The author suggests that practitioners may have worked the treatment and control groups quite differently; in addition to the differences necessitated by the deployment of FGDM
Differential diagnostic activity	Low risk	Data were harvested in retrospect from a social services database
Insensitive instrument used to measure	Unclear risk	Insufficient information is provided about how data from the social services database was coded
Researcher allegiance	Unclear risk	No information is provided about the authors’ links, or otherwise, to the intervention providers or funders. Authors do offer some appraisal of study limitations
Funding source	Unclear risk	No information provided
Contamination	High risk	The author describes the possibility of contamination bias

Berzin (2006): Fresno

**Table:** 

Methods	RCT
Participants	Children ages birth to 18 years who were assessed as being at moderate to high risk for further maltreatment and whose families were eligible for voluntary in‐home services
Interventions	Family Team Meetings (blends family unity and family group conference models)
Outcomes	Number of substantiated reports of maltreatment
	Impact on rate of removal from the home
	Placement stability
	Case closure for a positive reason
	Average time to permanency (case closure) for those case which were closed
Notes	

Risk of bias table

**Table:** 

Bias	Authors' judgement	Support for judgement
Random sequence generation (selection bias)	Unclear risk	Authors describe the study as a randomised controlled trial. A ratio of three treatment allocations to two control allocations is described. No further details of sequence generation are provided
Allocation concealment (selection bias)	Unclear risk	Insufficient information
Blinding of participants and personnel (performance bias)	High risk	Both study participants and practitioners were aware that they were involved in either the treatment or control arm of the experiment
Blinding of outcome assessment (detection bias)	High risk	Authors make no reference to the blinding of outcome assessors
Incomplete outcome reporting	High risk	A study protocol is not referenced. The narrative summary provided omits key data such as effect sizes, and variance measures. Data were only available from one of the two counties involved in the trial, for measures of placement permanency
Attrition bias	Unclear risk	Insufficient information
Study design	Unclear risk	The use of “sibling data” to inflate sample sizes, followed by the use of general estimating equations to counteract clustering effects, and the use of a fixed effects model, could be argued to be inappropriate analyses
Baseline imbalance	Unclear risk	A significantly higher number of children in the treatment group were female. Descriptive statistics for the samples at baseline are not provided
Confounding variable	High risk	The author suggests that practitioners may have worked the treatment and control groups quite differently; in addition to the differences necessitated by the deployment of FGDM
Differential diagnostic activity	Low risk	Data were harvested in retrospect from a social services database
Insensitive instrument used to measure	Unclear risk	Insufficient information is provided about how data from the social services database was coded
Researcher allegiance	Unclear risk	No information is provided about the authors’ links, or otherwise, to the intervention providers or funders. Authors do offer some appraisal of study limitations
Funding source	Unclear risk	No information provided
Contamination	High risk	The author describes the possibility of contamination bias

Berzin (2006): Riverside

**Table:** 

Methods	RCT
Participants	Children ages birth to 18 years who were assessed as being at moderate to high risk for further maltreatment and whose families were eligible for voluntary in‐home services
Interventions	Family Team Meetings (blends family unity and family group conference models)
Outcomes	Number of substantiated reports of maltreatment
	Impact on rate of removal from the home
	Placement stability
	Case closure for a positive reason
	Average time to permanency (case closure) for those case which were closed
Notes	

Risk of bias table

**Table:** 

Bias	Authors' judgement	Support for judgement
Random sequence generation (selection bias)	Unclear risk	Authors describe the study as a randomised controlled trial. A ratio of three treatment allocations to two control allocations is described. No further details of sequence generation are provided
Allocation concealment (selection bias)	Unclear risk	Insufficient information
Blinding of participants and personnel (performance bias)	High risk	Both study participants and practitioners were aware that they were involved in either the treatment or control arm of the experiment
Blinding of outcome assessment (detection bias)	High risk	Authors make no reference to the blinding of outcome assessors
Incomplete outcome reporting	High risk	A study protocol is not referenced. The narrative summary provided omits key data such as effect sizes, and variance measures. Data were only available from one of the two counties involved in the trial, for measures of placement permanency
Attrition bias	Unclear risk	Insufficient information
Study design	Unclear risk	The use of “sibling data” to inflate sample sizes, followed by the use of general estimating equations to counteract clustering effects, and the use of a fixed effects model, could be argued to be inappropriate analyses
Baseline imbalance	Unclear risk	A significantly higher number of children in the treatment group were female. Descriptive statistics for the samples at baseline are not provided
Confounding variable	High risk	The author suggests that practitioners may have worked the treatment and control groups quite differently; in addition to the differences necessitated by the deployment of FGDM
Differential diagnostic activity	Low risk	Data were harvested in retrospect from a social services database
Insensitive instrument used to measure	Unclear risk	Insufficient information is provided about how data from the social services database was coded
Researcher allegiance	Unclear risk	No information is provided about the authors’ links, or otherwise, to the intervention providers or funders. Authors do offer some appraisal of study limitations
Funding source	Unclear risk	No information provided
Contamination	High risk	The author describes the possibility of contamination bias

Crampton and Jackson (2007)

**Table:** 

Methods	A retrospective parallel cohort study
Participants	Non‐White children who have had a substantiated CPS case, out‐of‐home placement and no sexual abuse
Interventions	Family Group Decision Making (New Zealand family group conferencing is referenced)
Outcomes	Substantiated re‐referrals
	Placement stability (number of placement moves)
	Reunification
Notes	

Risk of bias table

**Table:** 

Bias	Authors' judgement	Support for judgement
Random sequence generation (selection bias)	High risk	This was a retrospective parallel cohort design
Allocation concealment (selection bias)	High risk	Not achievable with this study method
Blinding of participants and personnel (performance bias)	Low risk	Study participants and practitioners were unlikely to have been aware of the study during the decision‐making process
Blinding of outcome assessment (detection bias)	Unclear risk	Authors make no reference to the blinding of outcome assessors
Incomplete outcome reporting	Unclear risk	A study protocol, in which data collection and analysis would have been described, is not referenced
Attrition bias	Unclear risk	No analysis provided
Study design	High risk	Data is provided for three groups which did not recieve an FGDM meeting: families who were not deemed appropriate for referral to FGDM; families who refused FGDM; and families for whom the child removal petition had been withdrawn. The control group is therefore likely to be inherently different to the intervention group
		The intervention group comprised of families who had an FGDM meeting (data are reported separately for those who built a plan through FGDM, and those who did not; these data are amalgamated for the current synthesis). If FGDM did not result in a plan for the family then the integrity of the intervention is in doubt
Baseline imbalance	Unclear risk	No analysis is provided
Confounding variable	Unclear risk	Insufficient information
Differential diagnostic activity	Low risk	No indication
Insensitive instrument used to measure	Unclear risk	Authors describe the use of case files to track outcomes: additional information about this process was needed
Researcher allegiance	High risk	Authors state, in the study report introduction, that children placed through FGDM meetings are more likely to remain with their family. But authors do not cite supporting research of rigour for this standpoint
Funding source	Unclear risk	Insufficient information
Contamination	Unclear risk	Insufficient information

Cunning and Bartlett (2006): region 1

**Table:** 

Methods	Retrospective cohort study
Participants	Children who have been referred to a child protection agency
Interventions	Family Group Conferencing
Outcomes	Reunification with family
	Continued maltreatment
	Placement stability
Notes	

Risk of bias table

**Table:** 

Bias	Authors' judgement	Support for judgement
Random sequence generation (selection bias)	High risk	This was a retrospective parallel cohort design
Allocation concealment (selection bias)	High risk	Not achievable with this study method
Blinding of participants and personnel (performance bias)	Low risk	Study participants and practitioners were unlikely to have been aware of the study during intervention
Blinding of outcome assessment (detection bias)	High risk	Authors make no reference to the blinding of outcome assessors
Incomplete outcome reporting	High risk	A study protocol is not referenced. There appears to be an emphasis on comparisons which show FGDM to have worked, alongside selective outcome reporting
Attrition bias	Unclear risk	Insufficient information
Study design	High risk	Measurement variables were chosen after data were examined. The potential for researchers to choose variables, which offer more flattering efficacy findings, exists
Baseline imbalance	High risk	The authors acknowledge that their matched comparison group, was not viable. The comparison data extracted for this review relates to FGDM referred cases only. A comparison is possible as only some of these cases actually had a conference. A key problem with the rigour of the study, even after employing this selective data extraction: we do not know why some cases went on to have an FGDM conference, and some did not
Confounding variable	Unclear risk	Insufficient information
Differential diagnostic activity	Low risk	There is no information to suggest that differential diagnostic activity occurred. Authors describe a data verification process, in which 20% of extracted data were checked against source case files
Insensitive instrument used to measure	Unclear risk	Insufficient information
Researcher allegiance	High risk	There appears to be an emphasis on comparisons which show FGDM to have worked, alongside selective outcome reporting. For example, see Figure 4 (p. 21) for example of the study report which emphasises a favourable outcome
Funding source	Unclear risk	Insufficient information
Contamination	Unclear risk	Insufficient information

Cunning (2006): region 2

**Table:** 

Methods	Retrospective cohort study
Participants	Children who have been referred to a child protection agency
Interventions	Family Group Conferencing
Outcomes	Reunification with family
	Continued maltreatment
	Placement stability
Notes	

Risk of bias table

**Table:** 

Bias	Authors' judgement	Support for judgement
Random sequence generation (selection bias)	High risk	This was a retrospective parallel cohort design
Allocation concealment (selection bias)	High risk	Not achievable with this study method
Blinding of participants and personnel (performance bias)	Low risk	Study participants and practitioners were unlikely to have been aware of the study during intervention
Blinding of outcome assessment (detection bias)	High risk	Authors make no reference to the blinding of outcome assessors
Incomplete outcome reporting	High risk	A study protocol is not referenced. There appears to be an emphasis on comparisons which show FGDM to have worked, alongside selective outcome reporting
Attrition bias	Unclear risk	Insufficient information
Study design	High risk	Measurement variables were chosen after data were examined. The potential for researchers to choose variables, which offer more flattering efficacy findings, exists
Baseline imbalance	High risk	The authors acknowledge that their matched comparison group, was not viable. The comparison data extracted for this review relates to FGDM referred cases only. A comparison is possible as only some of these cases actually had a conference. A key problem with the rigour of the study, even after employing this selective data extraction: we do not know why some cases went on to have an FGDM conference, and some did not
Confounding variable	Unclear risk	Insufficient information
Differential diagnostic activity	Low risk	There is no information to suggest that differential diagnostic activity occurred. Authors describe a data verification process, in which 20% of extracted data were checked against source case files
Insensitive instrument used to measure	Unclear risk	Insufficient information
Researcher allegiance	High risk	There appears to be an emphasis on comparisons which show FGDM to have worked, alongside selective outcome reporting. For example, see Figure 4 (p. 21) for example of the study report which emphasises a favourable outcome
Funding source	Unclear risk	Insufficient information
Contamination	Unclear risk	Insufficient information

Dijkstra et al. (2019)

**Table:** 

Methods	Randomised controlled trial
Participants	Families referred to child protection services
Interventions	Family Group Conferencing
Outcomes	Continued maltreatment
	Expedition of case processing
	Service user satisfaction
	Engagement with support services
Notes	

Risk of bias table

**Table:** 

Bias	Authors' judgement	Support for judgement
Random sequence generation (selection bias)	Low risk	Families were randomly assigned to the experimental and control groups using a computer generated sequence
Allocation concealment (selection bias)	Unclear risk	Insufficient information
Blinding of participants and personnel (performance bias)	Unclear risk	Practitioners and families were aware of the study and which group each family was assigned to
Blinding of outcome assessment (detection bias)	Unclear risk	Insufficient information
Incomplete outcome reporting	Low risk	A study protocol is referenced
Attrition bias	Low risk	There was some imbalance in study drop out across the two groups but it was compensated for using multiple imputation (Graham, 2009)
Study design	Low risk	Considerable depth in study reporting provided. A study protocol is referenced. The study design described largely adheres to accepted guidance on the conduct of randomised controlled trials on complex interventions
Baseline imbalance	Low risk	Baseline characteristics are appropriately reported. No significant differences were found
Confounding variable	Unclear risk	Treatment integrity may have been compromised given the high level of staff turnover during the study
Differential diagnostic activity	Low risk	Diagnostic activity is described appropriately, with no indication of differential treatment across the two groups
Insensitive instrument used to measure	Low risk	Appropriate rationales are provided for each of the measurements applied
Researcher allegiance	Low risk	Authors state that they have no potentially conflicting interests. The topic area and study results are reported in an objective way
Funding source	Low risk	The funding source is identified as the Dutch Organization for Health Research and Development. Reviewers agreed that this organisation was not likely to be invested in a standpoint on FGDM efficacy
Contamination	Unclear risk	Insufficient information

Edwards et al. (2006)/Pennell (2010)

**Table:** 

Methods	Retrospective cohort study
Participants	Children who had been removed from their family
Interventions	Family Team Meetings
Outcomes	Number of children placed in kinship care
	Number of agreed case‐plans which included a reunification‐type goal
	Number of children who had exited foster care within 6 months
	Number of children reunified with their family
Notes	

Risk of bias table

**Table:** 

Bias	Authors' judgement	Support for judgement
Random sequence generation (selection bias)	High risk	This was a retrospective parallel cohort design
Allocation concealment (selection bias)	High risk	Not achievable with this study method
Blinding of participants and personnel (performance bias)	Low risk	Study participants and practitioners were unlikely to have been aware of the study during intervention
Blinding of outcome assessment (detection bias)	High risk	Authors make no reference to the blinding of outcome assessors
Incomplete outcome reporting	Unclear risk	A study protocol is not referenced
Attrition bias	Unclear risk	Insufficient information
Study design	Low risk	Study design choices do not appear to have affected findings for intervention and control groups differentially
Baseline imbalance	High risk	Baseline differences are reported
Confounding variable	High risk	FGDM families are likely to have had the benefit of additional support from FGDM coordinators
Differential diagnostic activity	Low risk	Similar diagnostic activity appears to be applied to both the FGDM and comparison group
Insensitive instrument used to measure	Low risk	The accuracy of some of the outcome measures used, to indicate child safety and well‐being, is uncertain, but time‐to‐foster‐care exit could be deemed to reasonably sensitive and unequivocal
Researcher allegiance	Unclear risk	Insufficient information
Funding source	Unclear risk	The study was funded by the intervention provider.
Contamination	Unclear risk	Insufficient information

Hollinshead (2017)/Corwin et al. (2019)

**Table:** 

Methods	Randomised controlled trial
Participants	Families referred to child protection services
Interventions	Family group conferencing/Ohana conferencing model
Outcomes	Substantiated re‐referrals
	Case‐workers' perceptions of social support
Notes	

Risk of bias table

**Table:** 

Bias	Authors' judgement	Support for judgement
Random sequence generation (selection bias)	Unclear risk	No details of the random sequence generation are provided
Allocation concealment (selection bias)	Unclear risk	Insufficient information
Blinding of participants and personnel (performance bias)	High risk	Practitioners and families were aware of the study and which group each family was assigned to
Blinding of outcome assessment (detection bias)	Unclear risk	Insufficient information
Incomplete outcome reporting	Low risk	A study protocol is referenced. All outcomes are reported
Attrition bias	Low risk	Intention‐to‐treat data were used
Study design	Low risk	No indications of potential study design biases across two study reports
Baseline imbalance	Low risk	Baseline comparisons were extensive and did not highlight differences
Confounding variable	Unclear risk	Data were gathered from casework staff. An unknown number of staff did not provide data. The families, which these staff worked with, were therefore precluded from study participation
Differential diagnostic activity	Low risk	Only 66% of caseworker questionnaires were returned. Authors acknowledged the potential for bias from this, and presented a Missing Completely at Random analysis which showed that the missing data were not systematic and affected both trial arms similarly
Insensitive instrument used to measure	Unclear risk	Case‐workers perceptions of social support could be argued to be an insensitive means of measuring children's risk of abuse and well‐being
Researcher allegiance	Low risk	A conflict of interest statement is provided. No conflicts of interest are provided. The study appears to be presented and reported in an objective way
Funding source	Low risk	Funding sources are declared: Children's Bureau, U.S. Department of Health and Human Services; and Casey Family Programs (U.S.)
Contamination	High risk	Authors point out that some families in the control group may have participated in FGCs. It is unclear if the same practitioners provided both FGC and non‐FGC case processing

McRae (2010)

**Table:** 

Methods	Retrospective parallel cohort study
Participants	Children who have been referred to a statutory child protection service
Interventions	Family group conferencing
Outcomes	Engagement in services (provided with the service, referred to the service, or service arranged)
Notes	

Risk of bias table

**Table:** 

Bias	Authors' judgement	Support for judgement
Random sequence generation (selection bias)	Unclear risk	This was a retrospective parallel cohort design
Allocation concealment (selection bias)	Unclear risk	Not achievable with this study method
Blinding of participants and personnel (performance bias)	Unclear risk	Study participants and practitioners were unlikely to have been aware of the study during the decision‐making process
Blinding of outcome assessment (detection bias)	Unclear risk	Authors make no reference to the blinding of outcome assessors
Incomplete outcome reporting	Unclear risk	A study protocol, in which data collection and analysis would have been described, is not referenced
Attrition bias	Unclear risk	Insufficient information
Study design	Low risk	The study draws on the 2001 US‐based National Survey of Child and Adolescent Well‐being (NSCAW) data. Infants and children investigated for sexual abuse were over‐sampled for the NSCAW
Baseline imbalance	High risk	Authors completed a comprehensive analysis of baseline characteristics and identified several significant differences between groups at baseline
Confounding variable	Unclear risk	No reference is made to possible confounders such as the reason for FGC referrals. Black children and white children were found to be referred to FGC at different rates. Treatment integrity is not discussed and is likely to be highly variable given the wide variety of agencies involved
Differential diagnostic activity	Unclear risk	Diagnostic activity appeared to be similar for both FGDM and the comparison group
Insensitive instrument used to measure	Unclear risk	Engagement in services (provided with the service, referred to the service, or service arranged) is dependant on case‐worker recording. It is unclear how accurately this recording was carried out
Researcher allegiance	Low risk	The authors presented findings objectively. The authors are employed at a University. Potential conflicts of interest are not discussed in the study report
Funding source	Unclear risk	The authors describe an appropriate funding source
Contamination	Unclear risk	Insufficient information

Pennell and Burford (2000)

**Table:** 

Methods	Prospective parallel cohort study
Participants	Children exposed to domestic violence, and at risk of removal from the home by child protection services
Interventions	Family Group Conferencing
Outcomes	Continued maltreatment (number of emergency removals of children from the home; and substantiated reports of maltreatment)
	Child well‐being (number of children self‐harming; number of children attempting to take their own life)
	Score from a domestic violence assessment tool
Notes	

Risk of bias table

**Table:** 

Bias	Authors' judgement	Support for judgement
Random sequence generation (selection bias)	High risk	Researchers requested that the most difficult cases be referred for FGDM
Allocation concealment (selection bias)	High risk	Not achievable with this study method
Blinding of participants and personnel (performance bias)	Low risk	Study participants and practitioners were unlikely to have been aware of the study during intervention
Blinding of outcome assessment (detection bias)	High risk	Authors make no reference to the blinding of outcome assessors
Incomplete outcome reporting	High risk	A number of findings are reported for FGDM only. A study protocol is not referenced
Attrition bias	Unclear risk	Insufficient information
Study design	High risk	Qualitative data is cited as evidence of efficacy (alongside quantitative findings)
Baseline imbalance	High risk	For example, child protection events at baseline: FGDM group had 233 events, the comparison had 129 events
Confounding variable	Unclear risk	Insufficient information
Differential diagnostic activity	High risk	Data from case files nad home visits appears to have been gathered in different ways for the two groups
Insensitive instrument used to measure	Unclear risk	A child protection events checklist was employed “culled from relevant literature” (p. 139): additional information was needed
Researcher allegiance	High risk	Authors argue for the benefits of FGDM throughout the article without reference to supporting evidence of rigour
Funding source	Unclear risk	Insufficient information
Contamination	Unclear risk	Insufficient information

Sundell and Vinnerljung (2004)

**Table:** 

Methods	A prospective cohort study
Participants	Children involved in child protection services
Interventions	Family Group Conferencing
Outcomes	Number of case closures
	Number of case re‐openings
	Number of re‐referrals
	Rate of placement in foster care
	Time spent in out‐of‐home care
	Proportion of out‐of‐home placements that were with relatives
Notes	

Risk of bias table

**Table:** 

Bias	Authors' judgement	Support for judgement
Random sequence generation (selection bias)	High risk	This was a parallel cohort prospective study with a nonequivalent comparison group
Allocation concealment (selection bias)	High risk	Not achievable with this study method
Blinding of participants and personnel (performance bias)	High risk	Participants self‐selected to a large degree, and social workers decided who was to be offered FGDM
Blinding of outcome assessment (detection bias)	Low risk	Outcome coders were blinded
Incomplete outcome reporting	Unclear risk	A study protocol is not referenced
Attrition bias	Unclear risk	Insufficient information. It is acknowledged that some attrition occurred, it is described as negligible, but details are not provided
Study design	High risk	Given the small sample size, it is very possible that individual social workers’ perspective may have affected results: social workers decided which families were to be offered FGDM. There was also a large degree of participant self‐selection
Baseline imbalance	High risk	Of all families referred to CPS during the study period, 35% were offered an FGC; only one in four of these families accepted the offer
Confounding variable	High risk	A higher proportion of the FGDM group were previously known to CPS (71% vs. 51%)
Differential diagnostic activity	Low risk	Authors report consideration of diagnostic issues, and measures to counteract these
Insensitive instrument used to measure	High risk	Authors acknowledge the imprecision of using re‐referrals as a measure of on‐going abuse
Researcher allegiance	Low risk	Both authors were based in government departments charged with research and evaluation of social services
Funding source	Low risk	Funding was provided by a government department charged with research and evaluation of social services
Contamination	High risk	Social workers from both FGDM and comparison groups were aware of FGDM provision ad involved in treatment allocation

Titcomb, Craig, and Lecroy (2005)

**Table:** 

Methods	Retrospective parallel cohort study
Participants	Children who have been referred to a statutory child protection service
Interventions	Family group decision making
Outcomes	Substantiated reports of continued abuse
Notes	

Risk of bias table

**Table:** 

Bias	Authors' judgement	Support for judgement
Random sequence generation (selection bias)	High risk	This was a retrospective parallel cohort design
Allocation concealment (selection bias)	High risk	Not achievable with this study method
Blinding of participants and personnel (performance bias)	Low risk	Study participants and practitioners were unlikely to have been aware of the study during the decision‐making process
Blinding of outcome assessment (detection bias)	High risk	Authors make no reference to the blinding of outcome assessors
Incomplete outcome reporting	Unclear risk	A study protocol, in which data collection and analysis would have been described, is not referenced
Attrition bias	Unclear risk	Insufficient information
Study design	Unclear risk	The bulk of this study is built on noncomparative analysis, that is, how FGDM performed against expectations. There is very limited information about comparative part of the study
Baseline imbalance	Unclear risk	Insufficient information
Confounding variable	Unclear risk	No reference is made to possible confounders such as the reason for FGDM referrals
Differential diagnostic activity	Low risk	Diagnostic activity appeared to be similar for both FGDM and the comparison group
Insensitive instrument used to measure	Unclear risk	More detailed information, was needed, about how the review period for the comparison group was constructed
Researcher allegiance	Unclear risk	The focus on findings of FGDM success, which are based on noncomparative analysis, might be construed as researcher allegiance bias. The lead author is based at a private consultancy service, arguably, it is difficult for such a company to offer a damning appraisal of any initiative they are contracted to evaluate
Funding source	Unclear risk	Funding sources are not adequately described
Contamination	High risk	Authors do not describe measures to counteract contamination bias. FGDM may have been delivered by workers who were also working on non‐FGDM cases

Walker (2005)

**Table:** 

Methods	Retrospective cohort study
Participants	Children involved in a child protection study
Interventions	Ohana Group Conferencing
Outcomes	Average number of times cases went to court
	Average number of times children were removed from care‐giver
	Average time to case closure
	NUmber of permanent custody orders
Notes	

Risk of bias table

**Table:** 

Bias	Authors' judgement	Support for judgement
Random sequence generation (selection bias)	High risk	This was a retrospective parallel cohort study. While 60 families were randomly selected from Department of Health records, there are no details of FGDM and comparison group allocation provided; authors do report that the number of cases reviewed was chosen by convenience
Allocation concealment (selection bias)	High risk	Not achievable with this study method
Blinding of participants and personnel (performance bias)	Low risk	Study participants and practitioners were unlikely to have been aware of the study during intervention
Blinding of outcome assessment (detection bias)	High risk	Authors make no reference to the blinding of outcome assessors
Incomplete outcome reporting	High risk	A study protocol is not referenced. Findings are very sparsely reported with key information missing
Attrition bias	Unclear risk	Insufficient information
Study design	Unclear risk	This was a study of pre‐existing data: no rationale for methods of data analysis are provided
Baseline imbalance	High risk	Authors note that there were baseline differences that could explain at least one of the outcomes of interest
Confounding variable	Unclear risk	Insufficient information
Differential diagnostic activity	Low risk	This was a study of pre‐existing data (case‐files). There is no indication that researchers employed different procedures for FGDM and comparison data
Insensitive instrument used to measure	High risk	Number of permanent custody orders, and average number of shelter placements are reported These are unlikely to be rigorous measures of efficacy given the small sample size
Researcher allegiance	Unclear risk	The author is an independent public health educator and lawyer in Waialua, Hawaii. It is unclear how this study was commissioned
Funding source	Unclear risk	Insufficient information
Contamination	Unclear risk	Insufficient information

Wang (2012)

**Table:** 

Methods	Retrospective cohort study
Participants	Children who had been placed in care for longer than a three‐day period
Interventions	Ohana Group Conferencing
Outcomes	Number of children in permanent placements at 15 months
	Number of children reunified with their family or placed with relatives at 18 months
Notes	

Risk of bias table

**Table:** 

Bias	Authors' judgement	Support for judgement
Random sequence generation (selection bias)	High risk	This was a secondary analysis of existing data
Allocation concealment (selection bias)	High risk	Not achievable with this study method
Blinding of participants and personnel (performance bias)	Low risk	Study participants and practitioners were unlikely to have been aware of the study during intervention
Blinding of outcome assessment (detection bias)	High risk	Authors make no reference to the blinding of outcome assessors
Incomplete outcome reporting	Unclear risk	A study protocol is not referenced. The first author provided all additional data requested through personal correspondence
Attrition bias	Unclear risk	Insufficient information
Study design	Low risk	Researchers published a discrete time survival analysis of pre‐existing data, and revisited the data to extract findings for the purposes of this review
Baseline imbalance	Unclear risk	Insufficient information
Confounding variable	Unclear risk	Insufficient information
Differential diagnostic activity	Low risk	Diagnostic activity was very straightforward
Insensitive instrument used to measure	Unclear risk	Findings provided compare FGDM and comparison group outcomes at 15 months, it is unclear if findings would have differed significantly if a different time period had been chosen
Researcher allegiance	Unclear risk	The researcher is likely to have worked closely with the agency responsible for the delivery of FGDM. Several authors worked for the agency
Funding source	Unclear risk	This study was conducted by the corresponding author under a contract between Texas Tech University and the Texas Department of Family and Protective Services
Contamination	Unclear risk	Insufficient information

Weigensberg (2009)

**Table:** 

Methods	Retrospective cohort study
Participants	Children who had contact with welfare services, but who were be cared for at home at baseline. Children were also aged 15 years and below, and had participated in the National Survey of Child and Adolescent Well‐Being (NSCAW, United States)
Interventions	Authors describe the key principles of FGDM, but do not offer details of the version of FGDM employed
Outcomes	Provision of, and engagement with, parent, child and family services
Notes	

Risk of bias table

**Table:** 

Bias	Authors' judgement	Support for judgement
Random sequence generation (selection bias)	High risk	This was a secondary analysis of existing data
Allocation concealment (selection bias)	High risk	Not achievable with this study method
Blinding of participants and personnel (performance bias)	Low risk	Study participants and practitioners were unlikely to have been aware of the study during intervention
Blinding of outcome assessment (detection bias)	High risk	Authors make no reference to the blinding of outcome assessors
Incomplete outcome reporting	High risk	A study protocol is not referenced. Some outcome data were unavailable
Attrition bias	Unclear risk	Insufficient information
Study design	High risk	The use of FGDM was not adequately confirmed. Survey data, from case workers, had to show that one family member was present for one FGDM meeting
Baseline imbalance	High risk	Propensity score matching was employed to eliminate most baseline imbalances. Authors point out that participant self‐selection was still likely
Confounding variable	Unclear risk	Insufficient information
Differential diagnostic activity	Unclear risk	Insufficient information
Insensitive instrument used to measure	High risk	Study authors, and review authors, questioned the validity of service engagement as a measure of FGDM efficacy
Researcher allegiance	Low risk	The authors offered a comprehensive analysis of potential study limitations. The authors reported findings objectively
Funding source	Unclear risk	Insufficient information
Contamination	Unclear risk	Insufficient information

Weisz (2006)

**Table:** 

Methods	Retrospective cohort study
Participants	Children who had been removed from their home
Interventions	Expedited family group conferencing
Outcomes	Number of removals during the evaluation period
	Number of placements
	Type of most recent placement
Notes	

Risk of bias table

**Table:** 

Bias	Authors' judgement	Support for judgement
Random sequence generation (selection bias)	High risk	This was a secondary analysis of existing data
Allocation concealment (selection bias)	High risk	Not achievable with this study method
Blinding of participants and personnel (performance bias)	Low risk	Study participants and practitioners were unlikely to have been aware of the study during intervention
Blinding of outcome assessment (detection bias)	High risk	Authors make no reference to the blinding of outcome assessors
Incomplete outcome reporting	High risk	A study protocol is not referenced. Some outcome data were unavailable
Attrition bias	Unclear risk	Insufficient information. Table 8 highlights missing data but there is no explanation of how this was accommodated
Study design	Unclear risk	In particular, insufficient information is provided about how the FGDM and comparison groups were selected
Baseline imbalance	High risk	Missing demographic data in the original dataset made controlling for baseline imbalance impossible
Confounding variable	Unclear risk	Insufficient information
Differential diagnostic activity	Unclear risk	Insufficient information
Insensitive instrument used to measure	High risk	“Time to discharge” and “final placement type” appear to be appropriate outcome measures
Researcher allegiance	Unclear risk	Insufficient information
Funding source	Unclear risk	Insufficient information
Contamination	Unclear risk	Insufficient information

**Characteristics of excluded studies**

**Table:** 

Aguiniga, Madden and Hawley (2015)	
Reason for exclusion	Wrong study design
Anderson (2003)	
Reason for exclusion	No comparison group
Anderson and Whalen, (2003)	
Reason for exclusion	Wrong study design
Antle, Barbee, Christensen and Sullivan (2009)	
Reason for exclusion	Wrong intervention
Reason for exclusion	Wrong outcomes
Baldry, Bratel, Dunsire and Durrant (2005)	
Reason for exclusion	Wrong population
Barlow et al. (2013)	
Reason for exclusion	Wrong population
Bell and Wilson (2006)	
Reason for exclusion	Wrong study design
Bribitzer and Verdieck (1988)	
Reason for exclusion	Wrong intervention
Brody et al. (2012)	
Reason for exclusion	Wrong intervention
Bröning et al. (2014)	
Reason for exclusion	Wrong intervention
Burford (1999)	
Reason for exclusion	Wrong study design
Connell, Dishion, Yasui and Kavanagh (2007)	
Reason for exclusion	Wrong intervention
Constantino et al. (2001)	
Reason for exclusion	Wrong intervention
Crampton (2003)	
Reason for exclusion	Insufficient data
Crampton (2007)	
Reason for exclusion	Qualitative study
Crampton, Usher, Wildire, Webster and Cuccaro‐Alamin (2011)	
Reason for exclusion	Comparison was not a parrallel cohort
Dalrymple (2002)	
Reason for exclusion	Wrong study design
Danielson et al. (2012)	
Reason for exclusion	Wrong population
DeGarmo, Reid, Fetrow, Fisher and Antoine (2013)	
Reason for exclusion	Wrong intervention
DePanfilis and Dubowitz (2005)	
Reason for exclusion	Wrong study design
Dijkstra et al. (2016)	
Reason for exclusion	Signs of Safety, not FGDM, was deployed to a significant proportion of the intervention group.
Dobbin (2001)	
Reason for exclusion	Wrong comparator
Eaton, Whalen and Anderson (2007)	
Reason for exclusion	Wrong study design
Ethier, Couture, Lacharite and Gagnier (2000)	
Reason for exclusion	Wrong intervention
Frost (2014)	
Reason for exclusion	Wrong study design
Goldbeck, Laib‐Koehnemund and Fegert (2007)	
Reason for exclusion	Wrong intervention
Gonzales et al. (2012)	
Reason for exclusion	Wrong intervention
Gopalan et al. (2015)	
Reason for exclusion	Wrong study design
Greenbaum et al. (2015)	
Reason for exclusion	Wrong study design
Guterman et al. (2013)	
Reason for exclusion	Wrong intervention
Hendriks, Van der Schee and Blanken (2011)	
Reason for exclusion	Wrong patient population
Holland and O'Neill (2006)	
Reason for exclusion	Wrong study design
Jeong, McGarrell and Hipple (2012)	
Reason for exclusion	Wrong study population (criminal justice‐involved youths) wrong outcome measures
Jones and Finnegan (2004)	
Reason for exclusion	Wrong study design
Jouriles et al. (2010)	
Reason for exclusion	Wrong intervention
Kolko, Iselin and Gully (2011)	
Reason for exclusion	Wrong intervention
Lambert, Johnson, and Wang (2017)	
Reason for exclusion	Comparison group was not a parrallel cohort
Landsman, Boel‐Studt and Malone (2014)	
Reason for exclusion	Wrong intervention
Liddle, Hogue, JJoM and Therapy (2000)	
Reason for exclusion	Wrong intervention
Linares et al. (2015)	
Reason for exclusion	Wrong intervention
Lupton (2003)	
Reason for exclusion	Insufficient data
Madden and Aguiniga (2013)	
Reason for exclusion	Wrong intervention (meditation not FGDM, see page 19)
Malmberg‐Heimonen and Johansen (2014)	
Reason for exclusion	Adult population
Marsh (2003)	
Reason for exclusion	Wrong study design
Marts, Lee, McRoy and McCroskey (2008)	
Reason for exclusion	Wrong study design
McGarrell and Hipple (2007)	
Reason for exclusion	Child population not necessarily at risk of abuse or neglect (i.e. young offenders)
Meezan and O'Keefe (1998)	
Reason for exclusion	Wrong intervention
Morris and Connolly (2012)	
Reason for exclusion	Wrong study design
O Brien (2000)	
Reason for exclusion	No comparison group
Olson (2003)	
Reason for exclusion	Wrong study design
Onrust, Romijn and de Beer (2015)	
Reason for exclusion	Wrong target population (not exclusively children at risk)
Pennell (2006)	
Reason for exclusion	No comparison
Perry (2014)	
Reason for exclusion	The comparison group also recieved a version of FGC
Prince, Gear, Jones and Read (2005)	
Reason for exclusion	Wrong study d
Pugh (2002)	
Reason for exclusion	Qualitative study
Quinnett (2003)	
Reason for exclusion	Wrong study design
Rauktis et al. (2010)	
Reason for exclusion	Wrong study design
Robbins et al. (2011)	
Reason for exclusion	Wrong patient population
Rodrigo, Máiquez, Correa, Martín and Rodríguez (2006)	
Reason for exclusion	Wrong intervention (community centre‐based program)
Rybski (1999)	
Reason for exclusion	Wrong intervention
Sieppert, Hudson and Unrau (2000)	
Reason for exclusion	No comparison group
Smith and Efron (2005)	
Reason for exclusion	Wrong intervention
Strong Families (2012)	
Reason for exclusion	Wrong study design
Swain and Ban (1997)	
Reason for exclusion	No comparison group
Taylor, Davis and Kemper (1997)	
Reason for exclusion	Wrong patient population
Teal (2013)	
Reason for exclusion	No comparison group (see page 33)
Thurston (2016)	
Reason for exclusion	Insufficient data
Walker (2010)	
Reason for exclusion	Wrong study design
Walton, Roby, Frandsen and Davidson (2004)	
Reason for exclusion	No comparison group
Wheeler (2003)	
Reason for exclusion	Wrong study design
Wijnen‐Lunenberg (2008)	
Reason for exclusion	Outcome was “average number of points of concern”; these were not exclusively indicators of maltreatment or neglect

## DATA AND ANALYSES

1. **Traditional child protection case processing**

**Table:** 

Outcome or Subgroup	Studies	Participants	Statistical method	Effect estimate
1.1 1.1Reunification of children with families or maintenance of in‐home care	10	86305	Odds Ratio (M‐H, Random, 95% CI)	1.69 [1.03, 2.78]
1.2 Continued maltreatment: effect sizes from nonrandomised studies	5	1779	Odds Ratio (M‐H, Random, 95% CI)	0.73 [0.48, 1.11]
1.3 Continued maltreatment:effect sizes from RCTs	4	1158	Odds Ratio (M‐H, Fixed, 95% CI)	1.29 [0.85, 1.98]
1.4 Kinship placements	5	85537	Odds Ratio (M‐H, Random, 95% CI)	1.29 [0.94, 1.76]

## SOURCES OF SUPPORT

### Internal sources

- No sources of support provided

### External sources

- No sources of support provided

OTHER REFERENCESAdditional references

Aber, J. L.
, 
Bennett, N. G.
, 
Conley, D. C.
, & 
Li, J.
 (1997). The effects of poverty on child health and development. Annual Review of Public Health, 18(1), 463–483.10.1146/annurev.publhealth.18.1.4639143727

Adams, P
, & 
Chandler, S. M.
 (2004). Responsive regulation in child welfare: Systemic challenges to mainstreaming the family group conference. Journal of Sociology and Social Welfare, 31, 93.

Barnsdale, L.
, & 
Walker, M
. (2007). *Examining the use and impact of Family Group Conferencing*. Edinburgh: Scottish Executive Edinburgh.

Bartlett, J. D.
, 
Kotake, C.
, 
Fauth, R.
, & 
Easterbrooks, M. A.
 (2017). Intergenerational transmission of child abuse and neglect: Do maltreatment type, perpetrator, and substantiation status matter?
Child Abuse and Neglect, 63, 84–94.2791423810.1016/j.chiabu.2016.11.021

Berliner, L.
 (2005). The results of randomized clinical trials move the field forward. Child Abuse and Neglect, 29(2), 103–105.1573417710.1016/j.chiabu.2004.08.007

Berzin, S. C.
 (2006). Using sibling data to understand the impact of family group decision‐making on child welfare outcomes. Children and Youth Services Review, 28(12), 1449–1458.

Berzin, S. C.
, 
Thomas, K. L.
, & 
Cohen, E.
 (2007). Assessing model fidelity in two family group decision‐making programs: Is this child welfare intervention being implemented as intended?
Journal of Social Service Research, 34(2), 55–71.

Burford, G.
 (1999). Letting the family speak about violence: Research findings on family group conference use in domestic violence. Child Care in Practice, 5(4), 350–360.
Campbell_Collaboration
. (2016). Methodological expectations of Campbell Collaboration intervention reviews: Reporting standards. Available from https://campbellcollaboration.org/meccir.html


Connolly, M.
, & 
MacKenzie, M.
 (1998). Effective participatory practice: Family group conferencing in child protection. London: Transaction Publishers.

Connolly, M.
, 
Crichton‐Hill, Y.
, & 
Ward, T.
 (2006). Culture and child protection: Reflexive responses. London: Jessica Kingsley Publishers.

Connolly, M.
 (2005). Fifteen years of family group conferencing: Coordinators talk about their experiences in Aotearoa New Zealand. British Journal of Social Work, 36(4), 523–540.

Crampton, D.
 (2003). Family group decision making in Kent County, Michigan: The family and community compact. Protecting Children, 18, 81–83.

Crampton, D.
 (2006). When do social workers and family members try family group decision making? A process evaluation. International Journal of Child and Family Welfare, 9(3), 131.

Crampton, D.
 (2007). Research review: Family group decision‐making: A promising practice in need of more programme theory and research. Child & Family Social Work, 12(2), 202–209.

Crampton, D.
, & 
Jackson, W. L.
 (2007a). Family group decision making and disproportionality in foster care: A case study. Child Welfare, 86(3), 51.17722681

Creemers, H. E.
, 
Sundell, K.
, 
Deković, M.
, 
Dijkstra, S.
, 
Stams, G. J. J.
, & 
Asscher, J. J.
 (2016). When the “Golden” standard should be the general standard: Response to a commentary on the use of randomised controlled trials to examine the effectiveness of family group conferencing. British Journal of Social Work, 47(4), 1262–1267.

Doolan, M.
 (2007). The response of law, policy and practice to participation rights in child welfare systems. Protecting Children, 22(1), 10–18.

Dwyer, L. A.
, 
Hornsey, M. J.
, 
Smith, L. G.
, 
Oei, T. P.
, & 
Dingle, G. A.
 (2011). Participant autonomy in cognitive behavioral group therapy: An integration of self‐determination and cognitive behavioral theories. Journal of Social and Clinical Psychology, 30(1), 24–46.

Edwards, L.
, & 
Sagatun‐Edwards, I.
 (2007). Transition to group decision making in child protection cases: Obtaining better results for children and families. Juvenile and Family Court Journal, 58, 1–16.
For the Veterans Affairs Cooperative Study Group on Primary Care and Hospital Readmission
. (1996). Does increased access to primary care reduce hospital readmissions. New England Journal of Medicine, 334(22), 1441–1447.10.1056/NEJM1996053033422068618584

Frost, N.
, 
Abram, F.
, & 
Burgess, H.
 (2014). Family group conferences: Evidence, outcomes and future research. Child & Family Social Work, 19(4), 501–507.

Graham, J.
 (2009). Missing data analysis: Making it work in the real world. Annual Review of Psychology, 2009(60), 549–576.10.1146/annurev.psych.58.110405.08553018652544

Havnen, K
, & 
Christiansen, O.
 (2014). Knowledge review on Family Group Conferencing, experiences and outcomes. Regional Centre for Child and Youth Mental Health and Child Welfare (RKBU West) Uni Research Health, Norway.

Higgins, J.
, & 
Green, S.
 (2009). The Cochrane Collaboration, Version 5.

Higgins, J. P. T.
, 
Altman, D. G.
, 
Gotzsche, P. C.
, 
Juni, P.
, 
Moher, D.
, 
Oxman, A. D.
, … 
Sterne, J. A. C.
 (2011). The Cochrane Collaboration's tool for assessing risk of bias in randomised trials. BMJ, 343, d5928.2200821710.1136/bmj.d5928PMC3196245

Holland, S.
, & 
Rivett, M.
 (2006). “Everyone started shouting”: Making connections between the process of family group conferences and family therapy practice. British Journal of Social Work, 38(1), 21–38.

Horwitz, M.
, & 
Marshall, T.
 (2015). Family engagement in child protection social work. Journal of Family Social Work, 18(4), 288–301.

Jones, L.
, & 
Daly, D.
 (2004). *Family unity meetings: Practice, research, and instructional curricula*. Retrieved from https://www.oercommons.org/courseware/module/21434/overview


Karanicolas, P. J.
, 
Farrokhyar, F.
, & 
Bhandari, M.
 (2010). Blinding: Who, what, when, why, how?
Canadian Journal of Surgery, 53(5), 345.PMC294712220858381
American Humane Society
. (2019). Quick reference guide: Various approaches and models to engage the family group in child welfare decision making. Available from http://www.americanhumane.org/assets/pdfs/children/fgdm/quick-reference.pdf


Lambert, M. C.
, 
Johnson, L. E.
, & 
Wang, E. W.
 (2017). The impact of family group decision‐making on preventing removals. Children and Youth Services Review, 78(C), 89–92.

Li, M.
, 
D'arcy, C.
, & 
Meng, X.
 (2016). Maltreatment in childhood substantially increases the risk of adult depression and anxiety in prospective cohort studies: Systematic review, meta‐analysis, and proportional attributable fractions. Psychological Medicine, 46(4), 717–730.2670827110.1017/S0033291715002743

Littell, J. H.
 (2001). Client participation and outcomes of intensive family preservation services. Social Work Research, 25(2), 103–113.

Lo, C. K. M.
, 
Chan, K. L.
, & 
Ip, P.
 (2017). Insecure adult attachment and child maltreatment: A meta‐analysis. Trauma, Violence & Abuse, 67, 193–206.10.1177/152483801773057929333992

Lohrbach, S.
 (2003). Family Group Decision Making: A process reflecting partnership‐based practice. Protecting Children, 18(1–2), 12–15.

Lupton, C.
, & 
Nixon, P.
 (1999). Empowering practice?: A critical appraisal of the family group conference approach. Bristol: Policy Press.

Lupton, C. S. M.
 (2003). Family outcomes: Following through on family group conferences. Protecting Children, 18(1 & 2), 127–129.

Marsh, P.
, & 
Crow, G.
 (1998). Family group conferences in child welfare. Oxford: Blackwell Science.

Merkel‐Holguin, L.
 (2003). Promising results, potential new directions: International FGDM research and evaluation in child welfare. Protecting Children, 18(1 & 2), 1–2.

Merkel‐Holguin, L.
, 
Nixon, P.
, & 
Burford, G.
 (2003). Learning with families: A synopsis of FGDM research and evaluation in child welfare. Protecting Children, 18, 2–12.

Morris, K.
, & 
Connolly, M.
 (2012). Family decision making in child welfare: Challenges in developing a knowledge base for practice. Child Abuse Review, 21(1), 41–52.

Nyberg, E.
 (2003). Family Group Conferencing in Sweden. Protecting Children, 18, 119–120.
Partnership for Strong Families
. (2009). *Engaging families through family team conferencing replication manual*. Retrieved from http://www.nrcpfc.org/grantees_public/2013/Partnership%20for%20Strong%20Families%20Replication%20Manual.pdf


Pennell, J.
, & 
Burford, G.
 (2000). Family group decision making: Protecting children and women. Child Welfare, 79(2), 131–158.10732256

Pennell, J.
, 
Edwards, M.
, & 
Burford, G.
 (2010). Expedited family group engagement and child permanency. Children and Youth Services Review, 32(7), 1012–1019.

Rauktis, M. E.
, 
McCarthy, S.
, 
Krackhardt, D.
, & 
Cahalane, H.
 (2010). Innovation in child welfare: The adoption and implementation of Family Group Decision Making in Pennsylvania. Children and Youth Services Review, 32(5), 732–739.

Rauktis, M. E.
, 
Bishop‐Fitzpatrick, L.
, 
Jung, N.
, & 
Pennell, J.
 (2013). Family group decision making: Measuring fidelity to practice principles in public child welfare. Children and Youth Services Review, 35(2), 287–95.

Robb, S. L.
, 
Burns, D. S.
, 
Docherty, S. L.
, & 
Haase, J. E.
 (2011). Ensuring treatment fidelity in a multi‐site behavioral intervention study: Implementing NIH behavior change consortium recommendations in the SMART trial. Psycho‐oncology, 20(11), 1193–1201.2201294310.1002/pon.1845PMC3198011

Rosenbaum, P. R.
, & 
Rubin, D. B.
 (1983). The central role of the propensity score in observational studies for causal effects. Biometrika, 70(1), 41–55.

Ryan, R. M.
, 
Lynch, M. F.
, 
Vansteenkiste, M.
, & 
Deci, E. L.
 (2011). Motivation and autonomy in counseling, psychotherapy, and behavior change: A look at theory and practice 1ψ7. The Counseling Psychologist, 39(2), 193–260.

Rychetnik, L.
, 
Frommer, M.
, 
Hawe, P.
, & 
Shiell, A.
 (2002). Criteria for evaluating evidence on public health interventions. Journal of Epidemiology and Community Health, 56(2), 119–127.1181281110.1136/jech.56.2.119PMC1732065

Schulz, K. F.
, & 
Grimes, D. A.
 (2002). Blinding in randomised trials: Hiding who got what. The Lancet, 359(9307), 696–700.10.1016/S0140-6736(02)07816-911879884

Sheets, J.
, 
Wittenstrom, K.
, 
Fong, R.
, 
James, J.
, 
Tecci, M.
, 
Baumann, D. J.
, & 
Rodriguez, C.
 (2009). Evidence‐based practice in family group decision‐making for Anglo, African American and Hispanic families. Children and Youth Services Review, 31(11), 1187–1191.

Shlonsky, A.
, 
Schumaker, K.
, 
Cook, C.
, 
Crampton, D.
, 
Saini, M.
, 
Backe‐Hansen, E.
, & 
Kowalski, K.
 (2009). Family group decision making for children at risk of abuse and neglect. Campbell Collaboration, 10, 14651858.

Shore, N.
, 
Wirth, J.
, 
Cahn, K.
, 
Yancey, B.
, & 
Gunderson, K.
 (2002). Long term and immediate outcomes of family group conferencing in Washington State. EFORUM. International Institute for Restorative Practices.

Sterne, J. A.
, 
Hernán, M. A.
, 
Reeves, B. C.
, 
Savović, J.
, 
Berkman, N. D.
, 
Viswanathan, M.
, … 
Higgins, J. P.
 (2016). ROBINS‐I: A tool for assessing risk of bias in non‐randomised studies of interventions. BMJ, 355, i4919.2773335410.1136/bmj.i4919PMC5062054

Sundell, K.
, & 
Vinnerljung, B.
 (2004). Outcomes of family group conferencing in Sweden: A 3‐year follow‐up. Child Abuse and Neglect, 28(3), 267–287.1506634610.1016/j.chiabu.2003.09.018

Tukey, J. W.
 (1986). Sunset salvo. The American Statistician, 40(1), 72–76.
UNICEF
Convention on the Rights of the Child. Available from https://www.unicef.org/child-rights-convention

UNICEF
. (2017). *A familiar face: Violence in the lives of children and adolescents*. Retrieved from https://www.unicef.org/publications/files/Violence_in_the_lives_of_children_Key_findings.pdf


Vesneski, W.
 (2009). Street‐level bureaucracy and family group decision making in the USA. Child & Family Social Work, 14(1), 1–5.

Walker, L.
 (2005). A cohort study of 'Ohana conferencing in child abuse and neglect cases. Protecting Children, 19(4), 36–46.

Wang, E. W.
, 
Lambert, M. C.
, 
Johnson, L. E.
, 
Boudreau, B.
, 
Breidenbach, R.
, & 
Baumann, D.
 (2012). Expediting permanent placement from foster care systems: The role of family group decision‐making. Children and Youth Services Review, 34(4), 845–850.

Weigensberg, E. C.
, 
Barth, R. P.
, & 
Guo, S.
 (2009). Family group decision making: A propensity score analysis to evaluate child and family services at baseline and after 36‐months. Children and Youth Services Review, 31(3), 383–390.

Weinberger, M.
, 
Oddone Eugene, Z.
, & 
Henderson William, G.
 (1996). Does increased access to primary care reduce hospital readmissions?
New England Journal of Medicine, 334(22), 1441–1447.10.1056/NEJM1996053033422068618584

White, H.
, & 
Sabarwal, S.
 (2014). Quasi‐experimental design and methods. Methodological briefs: Impact evaluation No. 8.
WHO
. (2016). *Child maltreatment*. Retrieved from http://www.who.int/mediacentre/factsheets/fs150/en/


Wick, E.
 (2014). *Family Group Decision Making: From Research to Application*. Supreme Court of Pennsylvania. Retrieved from http://www.ocfcpacourts.us/assets/upload/Resources/Documents/1D%20From%20Research%20to%20Application(1).pdf


Yatchmenoff, D. K.
 (2005). Measuring client engagement from the client's perspective in nonvoluntary child protective services. Research on Social Work Practice, 15(2), 84–96.

## APPENDIX A CRITERIA FOR THE RISK OF BIAS ASSESSMENTS

**Table:**

Sequence generation	Studies are to be rated as **low** risk if they adopted a typical method of random group assignment (for example using computer‐generated random number lists)
	Studies using comparison groups which may represent a population sub‐set are to be rated as **high** risk in this bias category
	An **unclear** risk is to be assigned were primary study authors failed to provide sufficient information. Or if they have used a comparison group which is likely to represent the study population with equivalence to the intervention group
Allocation sequence concealment	Randomised trials which concealed the sequence by which study participants were to be allocated, between FGDM and control intervention, from those in charge of study participant allocation were rated as **low** risk
	Nonrandomised studies are to be rated as having a **high** risk in this category
	Randomised trials which did not describe a means of allocation sequence concealment are to be rated with an **unclear** risk
Blinding of participants and personnel	If practitioners were not aware of research study (as is likely with retrospective parallel cohort studies) then the risk should be recorded as **low**
	If practitioners and participants, involved in delivering and receiving FGDM or the control intervention, were aware that the interventions were under study then the risk is to be recorded as **high**
	If it is not clear whether practitioners and participants were aware or not then the risk should be recorded as **unclear**
Blinding of outcome assessment	If a description of how outcome assessors were blinded is provided then the risk of bias should be recorded as **low**
	Risk of bias should be recorded as **high** if those charged with outcome data collection were privy to participant allocation between FGDM and the control intervention
	If no information regarding the blinding of outcome assessors is available then the risk of bias should be recorded as **unclear**
Incomplete outcome data	This should be recorded as **low**, if all data which plausibly should be reported, is reported, and all study participants are adequately accounted for in the reporting of findings, including data pertaining to participant withdrawals are refusals. In particular, any group differences in which participant withdrawals are refusals, should be described
	If there is a discrepancy between study participant numbers and reported outcome data then risk of bias should be recorded as **high**
	If data is missing due to participant withdrawal refusal to take part in the risk of bias should be recorded as **unclear**. If outcome data is only partially reported, for example if a measure of significance is reported without an effect size, or an average is reported without an indicator of variance then risk of bias should be recorded as unclear
Selective outcome reporting	If sufficient information is provided about the outcome measures deployed, and reported study findings correspond to the outcome measures used, then the risk of bias should also be reported as **low**
	Risk of bias should be recorded as **high** if only a subset of the original outcomes measured and analysed in a study are fully reported, or if there is any evidence of selective reporting of data on subgroups
	Or if primary study authors use finally treated rather than intention‐to‐treat analyses, if they choose to analyse continuously measured variables categorically, or if categorical variables parameters have been chosen insensitively, then risk of bias should be recorded as **unclear**
Study design bias	Within the boundaries of the general study design (e.g., a retrospective parallel cohort study will not feature the randomisation of study participants) if study design choices do not appear to have affected findings for intervention and control groups differentially, bias risk should be recorded as **low**
	Where study design choices, for example having participating families self‐selecting to intervention and control groups, are likely to bring about group differences, or confounding factors bias risk should be recorded as **high**
	Where study design choices, such as the choice of time point for data collection, have clearly affected findings for intervention and control groups differentially, bias risk should be recorded as **unclear**
Baseline imbalance	Where baseline differences have been comprehensively assessed, reported and found to be insignificant the risk of bias should be reported as **low**
	Indicators of sampling bias, such as the recruitment of an FGDM group of children with less complex needs than a comparison group, should attract a **high** risk rating. Indicators of significant group differences such as differences in the severity of abuse experienced by children in each group at baseline should also attract a rating of high risk
	Where baseline differences have not been assessed or reported adequately the risk should be recorded as **unclear**
Differential diagnostic activity	Record a **low** risk where diagnostic activity is adequately described with no apparent differences in how it was performed with the FGDM and comparison groups
	Record a **high** risk of bias if different measures, timeframes or data collection methods are employed with the intervention and comparison groups
	Record an **unclear** risk if there is an insufficient description of how data were collected
Insensitive instrument used for measurement	Outcomes of interest such as family permanence, placement stability, and prevention of child maltreatment should be measured sensitively for a **low** risk rating
	Examples of insensitive instruments will include the use of rating scales which are unlikely to capture the full range of data available. Where this is the case, bias risk should be recorded as **high**
	Where insufficient information is provided about the measurement tools used, an **unclear** risk should be recorded
Researcher allegiance bias	Researcher allegiance may affect a researcher's actions, and the reporting of results
	Wilson, G. Terence; Wilfley, Denise E.; Agras, W. Stewart; Bryson, Susan W. (2017‐03‐31). "Allegiance Bias and Therapist Effects: Results of a Randomized Controlled Trial of Binge Eating Disorder". *Clinical Psychology*: a publication of the Division of Clinical Psychology of the American Psychological Association. 18(2):119–125. ISSN 0969[JW1]
	We would add that practitioners, who are invested in FGDM or control interventions, may advertently or inadvertently affect intervention delivery and outcome data collection
	Where no examples of researcher, or practitioner allegiance bias are found the risk should be reported as **low**
	Where there are explicit examples of this in the primary study report, for example, the inappropriate characterisation of findings, the risk should be recorded as **high**
	Less explicit examples should attract an **unclear** rating
Funding source bias	It is difficult to conceive an assessment of high risk of funding source bias in this field, however where the study has been funded by proponents of FGDM[JW2] [TM3], and steps to ensure researcher independence are not described, it may be appropriate to record a risk of **unclear**
	Where the funding source bears no plausible connection to the promotion of FGDM or control interventions this risk should be recorded as **low**
Contamination bias	The key principles of FGDM could conceivably be assimilated into traditional child protection service delivery. In general terms, it is acknowledged that FGDM has influenced the conduct of child protection work across the globe
	Where it is clear that the implementation of FGDM has been kept separate from traditional child protection services then a **low** risk should be recorded
	Explicit examples of the use of FGDM in control interventions will attract a **high** risk rating, as would the use of the same practitioners to deliver both interventions
	Where there is insufficient information provided to make a judgement on this risk, but it appears likely that the same practitioners were used for both FGDM and control interventions then on **unclear** risk should be recorded

## APPENDIX B EXCLUDED STUDIES

**Table:**

Aguiniga	2015	Wrong study design
Anderson	2004	No comparison group
Anderson and Whalen	2003	Wrong study design
Antle	2009	Wrong intervention
Appleton	2013	Wrong outcomes
Baldry	2005	Wrong patient population
Barlow	2013	Wrong patient population
Bell	2006	Wrong study design
Bribitzer	1988	Wrong intervention
Brody	2012	Wrong intervention
Broning	2014	Wrong intervention
Bryant	2010	Wrong intervention
Burford	2006	Wrong study design
Colman	2007	Wrong study design
Connell	2007	Wrong intervention
Constantino	2004	Wrong intervention (Urban Home Intervention)
Crampton	2011	Comparison was not a parallel cohort
Crampton	2001	Insufficient data
Crampton	2003	Insufficient data
Crampton	2007	Qualitative study
Dalrymple	2002	Wrong study design
Danielson	2012	Wrong patient population
DeGarmo	2013	Wrong intervention
DePanfilis	2005	Wrong study design
Dijsktra	2016	Signs of Safety, not FGDM, was deployed to a significant proportion of the intervention group
Dobbin	2001	Wrong comparator
Eaton	2007	Wrong study design
Edwards	2005	Insufficient data
Ethier	2000	Wrong intervention
Frost	2014	Wrong study design
Gatowski	2005	Wrong intervention (mediation not FGDM (see p. 28)
Ghasemi	2014	Wrong intervention
Goldbeck	2007	Wrong intervention
Gonzales	2012	Wrong intervention
Gopalan	2015	Wrong study design
Greenbaum	2015	Wrong study design
Guterman	2013	Wrong intervention
Hendriks	2011	Wrong patient population
Holland	2006	Wrong study design
Jeong	2012	Wrong study population (criminal justice‐involved youths) wrong outcome measures
Jones	2004	Wrong study design
Jouriles	2010	Wrong intervention (support project)
Kolko	2011	Wrong intervention
Lambert	2017	Comparison group was not a parallel cohort
Landsman	2014	Wrong intervention
Liddle	2000	Wrong intervention (family therapy)
Linares	2015	Wrong intervention
Lupton	1997	Insufficient data
Lupton	2003	Wrong study design
Madden	2013	Wrong intervention (meditation not FGDM, see p. 19)
Malmberg‐Heimonen	2014	Adult population
Marsh	2003	Wrong study design
Marts	2008	Wrong study design
McCrae	2010	Wrong study design
McGarrell	2007	Child population not necessarily at risk of abuse or neglect (i.e., young offenders)
Meezan	1998	Wrong intervention
Morris	2012	Wrong study design
O'Brien	2000	No comparison group
Olson	2003	Wrong study design
Onrust	2015	Wrong target population (not exclusively children at risk)
Pennell	2006	No comparison group
Perry	2014	The comparison group also received a version of FGDM
Prince	2005	Wrong study design
Pugh	2002	Qualitative study
Quinnett	2003	Wrong study design
Rauktis	2010	Wrong study design
Robbins	2011	Wrong patient population
Rodrigo	2006	Wrong intervention (community centre‐based program)
Rybski	1999	Wrong intervention
Sieppert	2000	No comparison group
Smith	2005	Wrong intervention
Strong Families	2012	Wrong study design
Swain	1997	No comparison group
Taylor	1997	Wrong patient population
Teal	2013	No comparison group (see page 33)
Thurston	2016	Study will not be completed before completion of current review
Titcomb	2005	Insufficient data
Walker	2010	Wrong study design
Walton	2004	No comparison group
Wheeler	2003	Wrong study design
Wijnen‐Lunenberg	2008	Outcome was “average number of points of concern”; these were not exclusively indicators of maltreatment or neglect
Wingrove	2005	The article/report was intractably unavailable, but study data is believed to be included in Weisz, V., Korpas, A., & Wingrove, T. (2006), which has been included in this review

## APPENDIX C SEARCH STRATEGIES

### SEARCH STRATEGIES

The original systematic literature search was carried out by librarians Tania Celeste & Frances Morrissey, Scholarly Information (Brownless Biomedical Library), University of Melbourne, in July 2016. Thirteen resources were searched: ASSIA, CINAHL, Embase, Eric, Subfiles of Evidence Based Medicine Reviews, Family, IBSS, Medline, NCJRS, ProQuest Dissertations & Theses Global, PsycInfo, SocIndex and Sociological Abstracts. The searches were broadly and substantively similar but leveraged controlled vocabularies and search operators unique to each resource. For example, the construction “random* control* trial” could not be used in ProQuest as the internal wildcards were not recognised. The first and second authors repeated these searches in August 2019, and filtered results by date to capture any additional relevant publications between July 2016 and August 2019. These searches produced 213 records for screening. Two of these records were found to satisfy study selection criteria, and correspondence with the authors of one of these study reports led to the acquisition of three additional study reports, relating to two additional studies. Search activity in 2019, therefore, led to the inclusion of four additional studies in the review.

### ASSIA ProQuest Search Strategy (21 July 2016)

**Table:**

Set#	Searched for	Results
S1	family group or family decision* or family conferenc* or family meeting or family unity or family team or family centred or family centered or FGC or FGDM	30,333^a^
S2	famil* or parent* or caregiver* or guardian*	105,039^a^
S3	group conference* or group decision* or team conference* or team decision* or case meeting or case planning or planning meeting or consensus‐based decision‐making or consensus based decision making or consensus based decisionmaking	15,605^a^
S4	abuse* or neglect* or maltreat*	44,069^a^
S5	SU.EXACT(“Child abuse”) OR SU.EXACT(“Abused children”)	2,642^a^
S6	(abuse* or neglect* or maltreat*) OR (SU.EXACT(“Child abuse”) OR SU.EXACT(“Abused children”))	44,069^a^
S7	SU.EXACT(“Children”) OR SU.EXACT(“Adolescents”)	56,063^a^
S8	((abuse* or neglect* or maltreat*) OR (SU.EXACT(“Child abuse”) OR SU.EXACT(“Abused children”))) AND (SU.EXACT(“Children”) OR SU.EXACT(”Adolescents”))	5,542^a^
S9	SU.EXACT(“Child welfare”)	1,210^a^
S10	child advocacy	636^a^
S11	SU.EXACT(“Social work”)	7,915^a^
S12	child protection	3,564^a^
S13	engag* or involv* or partnership or participat* or conferenc* or meeting* or mediat*	128,563^a^
S14	child protection conference* or child protection meeting* or child protection mediat*	186^a^
S15	(family group or family decision* or family conferenc* or family meeting or family unity or family team or family centred or family centered or FGC or FGDM) AND (((abuse* or neglect* or maltreat*) OR (SU.EXACT(“Child abuse”) OR SU.EXACT(“Abused children”))) AND (SU.EXACT(“Children”) OR SU.EXACT(“Adolescents”)))	683^a^
S16	(famil* or parent* or caregiver* or guardian*) AND (group conference* or group decision* or team conference* or team decision* or case meeting or case planning or planning meeting or consensus‐based decision‐making or consensus based decision making or consensus based decisionmaking) AND (((abuse* or neglect* or maltreat*) OR (SU.EXACT(“Child abuse”) OR SU.EXACT(“Abused children”))) AND (SU.EXACT("Children") OR SU.EXACT("Adolescents")))	53^a^
S17	SU.EXACT(“Child welfare”) OR (child advocacy) OR SU.EXACT(“Social work”) OR (child protection)	12,616^a^
S18	(famil* or parent* or caregiver* or guardian*) AND (((abuse* or neglect* or maltreat*) OR (SU.EXACT(“Child abuse”) OR SU.EXACT(“Abused children”))) AND (SU.EXACT(”Children”) OR SU.EXACT(“Adolescents”))) AND (engag* or involv* or partnership or participat* or conferenc* or meeting* or mediat*) AND (SU.EXACT(“Child welfare”) OR (child advocacy) OR SU.EXACT(“Social work”) OR (child protection))	172^a^
S19	(famil* or parent* or caregiver* or guardian*) AND (child protection conference* or child protection meeting* or child protection mediat*)	110^a^
S20	(random* control* trial* OR random* control* stud*) OR SU.EXACT(“Clinical randomized controlled trials” OR “Cluster randomized controlled trials” OR “Double blind randomized controlled trials” OR “Randomized consent design” OR “Randomized controlled trials” OR “Single blind randomized controlled trials” OR “Urn randomization”)	14,975^a^
S21	((family group or family decision* or family conferenc* or family meeting or family unity or family team or family centred or family centered or FGC or FGDM) AND (((abuse* or neglect* or maltreat*) OR (SU.EXACT(“Child abuse”) OR SU.EXACT“Abused children”))) AND (SU.EXACT(“Children”) OR SU.EXACT(“Adolescents”)))) OR ((famil* or parent* or caregiver* or guardian*) AND (group conference* or group decision* or team conference* or team decision* or case meeting or case planning or planning meeting or consensus‐based decision‐making or consensus based decision making or consensus based decisionmaking) AND (((abuse* or neglect* or maltreat*) OR (SU.EXACT(“Child abuse”) OR SU.EXACT(“Abused children”))) AND (SU.EXACT(“Children”) OR SU.EXACT(“Adolescents”)))) OR ((famil* or parent* or caregiver* or guardian*) AND (((abuse* or neglect* or maltreat*) OR (SU.EXACT(“Child abuse”) OR SU.EXACT(“Abused children”))) AND (SU.EXACT(“Children”) OR SU.EXACT(“Adolescents”))) AND (engag* or involv* or partnership or participat* or conferenc* or meeting* or mediat*) AND (SU.EXACT(“Child welfare”) OR (child advocacy) OR SU.EXACT(“Social work”) OR (child protection))) OR ((famil* or parent* or caregiver* or guardian*) AND (child protection conference* or child protection meeting* or child protection mediat*))	915^a^
S22	((random* control* trial* OR random* control* stud*) OR SU.EXACT(“Clinical randomized controlled trials” OR “Cluster randomized controlled trials” OR “Double blind randomized controlled trials” OR “Randomized consent design” OR “Randomized controlled trials” OR “Single blind randomized controlled trials” OR “Urn randomization”)) AND (((family group or family decision* or family conferenc* or family meeting or family unity or family team or family centred or family centered or FGC or FGDM) AND (((abuse* or neglect* or maltreat*) OR (SU.EXACT(“Child abuse”) OR SU.EXACT(“Abused children”))) AND (SU.EXACT(“Children”) OR SU.EXACT(“Adolescents”)))) OR ((famil* or parent* or caregiver* or guardian*) AND (group conference* or group decision* or team conference* or team decision* or case meeting or case planning or planning meeting or consensus‐based decision‐making or consensus based decision making or consensus based decisionmaking) AND (((abuse* or neglect* or maltreat*) OR (SU.EXACT(“Child abuse”) OR SU.EXACT(“Abused children”))) AND (SU.EXACT(“Children”) OR SU.EXACT(“Adolescents”)))) OR ((famil* or parent* or caregiver* or guardian*) AND (((abuse* or neglect* or maltreat*) OR (SU.EXACT(“Child abuse”) OR SU.EXACT(“Abused children”))) AND (SU.EXACT(“Children”) OR SU.EXACT(“Adolescents”))) AND (engag* or involv* or partnership or participat* or conferenc* or meeting* or mediat*) AND (SU.EXACT(“Child welfare”) OR (child advocacy) OR SU.EXACT(“Social work”) OR (child protection))) OR ((famil* or parent* or caregiver* or guardian*) AND (child protection conference* or child protection meeting* or child protection mediat*)))	50^a^

^a^ Duplicates were removed from the search and result count.

### IBSS ProQuest Search Strategy (22 July 2016)

**Table:**

Set#	Searched for	Results
S1	ab(“randomised controlled trial” OR “randomized controlled trial” OR “randomised controlled study” OR “randomized controlled study”)	432^a^
S2	ab((control* OR prospectiv*) N/10 (study or trial))	5,694^a^
S3	ab(random*)	12,963^a^
S4	ab(“clinical trial”)	296^a^
S5	ab((singl* or doubl* or trebl* or tripl*) N/10 (blind* or mask*))	157^a^
S6	S1 OR S2 OR (S3 AND S4) OR S5	5,885^a^
S7	ab(“family group” or “family decision*” or “family conferenc*” or “family meeting” or “family unity” or “family team” or “family centred” or “family centered” or FGC or FGDM)	346^a^
S8	ab(famil* or parent* or caregiver* or guardian*)	55,621^a^
S9	ab(“group conference*” or “group decision*” or “team conference*” or “team decision*” or “case meeting” or “case planning” or “planning meeting” or “consensus‐based decision‐making” or “consensus based decision making” or “consensus based decisionmaking”)	349^a^
S10	ab(abuse* or neglect* or maltreat*)	14,974^a^
S11	ab(child* OR adolescen* OR teenage* OR infant*)	47,362^a^
S12	ab(“child abuse”)	377^a^
S13	ab(“child welfare” OR “child advocacy” OR “child protection” OR “social work” OR “social services”)	5,116^a^
S14	ab(engag* or involv* or partnership or participat* or conferenc* or meeting* or mediat*)	141,627^a^
S15	ab(“child protection conference*” or “child protection meeting*” or “child protection mediat*”)	1^a^
S16	(S10 AND S11) OR S12	2,698^a^
S17	S7 AND S16	9^a^
S18	S8 AND S9 AND S16	2^a^
S19	S8 AND S13 AND S14 AND S16	88^a^
S20	S8 AND S15	1^a^
S21	(S17 OR S18 OR S19 OR S20) AND S6	2^a^

^a^ Duplicates were removed from the search and result count.

### NCJRS ProQuest Search Strategy (22 July 2016)

**Table:**

Set#	Searched for	Results
S1	ab(“randomised controlled trial” OR “randomized controlled trial” OR “randomised controlled study” OR “randomized controlled study”)	67^a^
S2	ab((control* OR prospectiv*) N/10 (study or trial))	3,410^a^
S3	ab(random*)	6,416^a^
S4	ab(“clinical trial”)	63^a^
S5	ab((singl* or doubl* or trebl* or tripl*) N/10 (blind* or mask*))	76^a^
S6	S1 OR S2 OR (S3 AND S4) OR S5	3,502^a^
S7	ab(“family group” or “family decision*” or “family conferenc*” or “family meeting” or “family unity” or "family team” or “family centred” or “family centered” or FGC or FGDM)	367^a^
S8	ab(famil* or parent* or caregiver* or guardian*)	36,128^a^
S9	ab(“group conference*” or “group decision*” or “team conference*” or “team decision*” or “case meeting” or “case planning” or “planning meeting” or "consensus‐based decision‐making" or “consensus based decision making” or “consensus based decisionmaking”)	286^a^
S10	ab(abuse* or neglect* or maltreat*)	32,446^a^
S11	ab(child* OR adolescen* OR teenage* OR infant*)	37,317^a^
S12	ab(“child abuse”)	5,077^a^
S13	ab(“child welfare” OR “child advocacy” OR “child protection” OR “social work” OR “social services”)	5,374^a^
S14	ab(engag* or involv* or partnership or participat* or conferenc* or meeting* or mediat*)	74,199^a^
S15	ab(“child protection conference*” or “child protection meeting*” or “child protection mediat*”)	9^a^
S16	(S10 AND S11) OR S12	15,842^a^
S17	S7 AND S16	71^a^
S18	S8 AND S9 AND S16	33^a^
S19	S8 AND S13 AND S14 AND S16	809^a^
S20	S8 AND S15	6^a^
S21	(S17 OR S18 OR S19 OR S20) AND S6	7^a^

^a^ Duplicates were removed from the search and result count.

### Sociological Abstracts ProQuest Search Strategy (21 July 2016)

**Table:**

	Search	Results
S22	(random* control* trial* OR random* control* stud*) AND (((family group or family decision* or family conferenc* or family meeting or family unity or family team or family centred or family centered or FGC or FGDM) AND (((abuse* or neglect* or maltreat*) OR SU.EXACT(“Child Abuse”)) AND (SU.EXACT(“Adolescents”) OR SU.EXACT(“Children”)))) OR ((famil* or parent* or caregiver* or guardian*) AND (group conference* or group decision* or team conference* or team decision* or case meeting or case planning or planning meeting or consensus‐based decision‐making or consensus based decision making or consensus based decisionmaking) AND (((abuse* or neglect* or maltreat*) OR SU.EXACT(“Child Abuse”)) AND (SU.EXACT(“Adolescents”) OR SU.EXACT(“Children”)))) OR ((famil* or parent* or caregiver* or guardian*) AND (((abuse* or neglect* or maltreat*) OR SU.EXACT(“Child Abuse”)) AND (SU.EXACT(“Adolescents”) OR SU.EXACT(“Children”))) AND (engag* or involv* or partnership or participat* or conferenc* or meeting* or mediat*) AND (SU.EXACT(“Child Welfare Services”) OR (child advocacy) OR SU.EXACT(“Social Work”) OR (child protection))) OR ((famil* or parent* or caregiver* or guardian*) AND (child protection conference* or child protection meeting* or child protection mediat*)))	34^a^
S21	random* control* trial* OR random* control* stud*	2,889^a^
S20	((family group or family decision* or family conferenc* or family meeting or family unity or family team or family centred or family centered or FGC or FGDM) AND (((abuse* or neglect* or maltreat*) OR SU.EXACT(“Child Abuse”)) AND (SU.EXACT(“Adolescents”) OR SU.EXACT(“Children”)))) OR ((famil* or parent* or caregiver* or guardian*) AND (group conference* or group decision* or team conference* or team decision* or case meeting or case planning or planning meeting or consensus‐based decision‐making or consensus based decision making or consensus based decisionmaking) AND (((abuse* or neglect* or maltreat*) OR SU.EXACT(“Child Abuse”)) AND (SU.EXACT(“Adolescents”) OR SU.EXACT(“Children”)))) OR ((famil* or parent* or caregiver* or guardian*) AND (((abuse* or neglect* or maltreat*) OR SU.EXACT(“Child Abuse”)) AND (SU.EXACT(“Adolescents”) OR SU.EXACT(“Children”))) AND (engag* or involv* or partnership or participat* or conferenc* or meeting* or mediat*) AND (SU.EXACT(“Child Welfare Services”) OR (child advocacy) OR SU.EXACT(“Social Work”) OR (child protection))) OR ((famil* or parent* or caregiver* or guardian*) AND (child protection conference* or child protection meeting* or child protection mediat*))	2,095^a^
S19	(famil* or parent* or caregiver* or guardian*) AND (child protection conference* or child protection meeting* or child protection mediat*)	173^a^
S18	(famil* or parent* or caregiver* or guardian*) AND (((abuse* or neglect* or maltreat*) OR SU.EXACT(“Child Abuse”)) AND (SU.EXACT(“Adolescents”) OR SU.EXACT(“Children”))) AND (engag* or involv* or partnership or participat* or conferenc* or meeting* or mediat*) AND (SU.EXACT(“Child Welfare Services”) OR (child advocacy) OR SU.EXACT(“Social Work”) OR (child protection))	215^a^
S17	SU.EXACT(“Child Welfare Services”) OR (child advocacy) OR SU.EXACT(“Social Work”) OR (child protection)	7,039^a^
S16	(famil* or parent* or caregiver* or guardian*) AND (group conference* or group decision* or team conference* or team decision* or case meeting or case planning or planning meeting or consensus‐based decision‐making or consensus based decision making or consensus based decisionmaking) AND (((abuse* or neglect* or maltreat*) OR SU.EXACT(“Child Abuse”)) AND (SU.EXACT(“Adolescents”) OR SU.EXACT(“Children”)))	125^a^
S15	(family group or family decision* or family conferenc* or family meeting or family unity or family team or family centred or family centered or FGC or FGDM) AND (((abuse* or neglect* or maltreat*) OR SU.EXACT(“Child Abuse”)) AND (SU.EXACT(“Adolescents”) OR SU.EXACT(“Children”)))	1,811^a^
S14	child protection conference* or child protection meeting* or child protection mediat*	247^a^
S13	engag* or involv* or partnership or participat* or conferenc* or meeting* or mediat*	248,994^a^
S12	child protection	2,684^a^
S11	SU.EXACT(“Social Work”)	2,593^a^
S10	child advocacy	681^a^
S9	SU.EXACT(“Child Welfare Services”)	1,970^a^
S8	((abuse* or neglect* or maltreat*) OR SU.EXACT(“Child Abuse”)) AND (SU.EXACT(“Adolescents”) OR SU.EXACT(“Children”))	7,040^a^
S7	SU.EXACT(“Adolescents”) OR SU.EXACT(“Children”)	44,888^a^
S6	(abuse* or neglect* or maltreat*) OR SU.EXACT(“Child Abuse”)	50,346^a^
S5	SU.EXACT(“Child Abuse”)	2,672^a^
S4	abuse* or neglect* or maltreat*	50,346^a^
S3	group conference* or group decision* or team conference* or team decision* or case meeting or case planning or planning meeting or consensus‐based decision‐making or consensus based decision making or consensus based decisionmaking	40,651^a^
S2	famil* or parent* or caregiver* or guardian*	202,454^a^
S1	family group or family decision* or family conferenc* or family meeting or family unity or family team or family centred or family centered or FGC or FGDM	76,352^a^

^a^ Duplicates were removed from the search and result count.

### ProQuest Dissertations and Theses Global Search Strategy (21 July 2016)

**Table:**

Set#	Searched for	Results
S1	ab(“randomised controlled trial” OR “randomized controlled trial” OR “randomised controlled study” OR “randomized controlled study”)	1,998^a^
S2	ab((control* OR prospectiv*) N/10 (study or trial))	65,981^a^
S3	ab(random*)	121,337^a^
S4	ab(“clinical trial”)	3,044^a^
S5	ab((singl* or doubl* or trebl* or tripl*) N/10 (blind* or mask*))	2,949^a^
S6	S1 OR S2 OR (S3 AND S4) OR S5	68,289^a^
S7	ab(“family group” or “family decision*” or “family conferenc*” or “family meeting” or “family unity” or “family team” or "family centred” or “family centered” or FGC or FGDM)	1,403^a^
S8	ab(famil* or parent* or caregiver* or guardian*)	281,239^a^
S9	ab(“group conference*” or “group decision*” or “team conference*” or “team decision*” or “case meeting” or “case planning” or “planning meeting” or "consensus‐based decision‐making" or “consensus based decision making” or “consensus based decisionmaking”)	1,174^a^
S10	ab(abuse* or neglect* or maltreat*)	51,473^a^
S11	ab(child* OR adolescen* OR teenage* OR infant*)	202,267^a^
S12	ab(“child abuse”)	2,039^a^
S13	ab(“child welfare” OR “child advocacy” OR “child protection” OR “social work” OR “social services”)	11,247^a^
S14	ab(engag* or involv* or partnership or participat* or conferenc* or meeting* or mediat*)	700,266^a^
S15	ab(“child protection conference*” or “child protection meeting*” or “child protection mediat*”)	9^a^
S16	(S10 AND S11) OR S12	14,437^a^
S17	S7 AND S16	49^a^
S18	S8 AND S9 AND S16	11^a^
S19	S8 AND S13 AND S14 AND S16	486^a^
S20	S8 AND S15	9^a^
S21	(S17 OR S18 OR S19 OR S20) AND S6	27^a^

^a^ Duplicates were removed from the search and result count.

### Family INFORMIT Search Strategy (20 July 2016)

**Table:**

Set#	Searched for	Results
27	((random* control* trial*) OR (random* control* stud*)) AND (((“child protection conferenc*” OR “child protection meeting*” OR “child protection mediation”) AND (famil* OR parent* OR caregiver*…	0
26	(random* control* trial*) OR (random* control* stud*)	155
21	((“child protection conferenc*” OR “child protection meeting*” OR “child protection mediation”) AND (famil* OR parent* OR caregiver* OR guardian*)) OR (((child protection) OR (social work) OR…	1,339
20	(“child protection conferenc*” OR “child protection meeting*” OR “child protection mediation”) AND (famil* OR parent* OR caregiver* OR guardian*)	0
19	((child protection) OR (social work) OR (child advocacy) OR (child welfare)) AND (engag* OR involv* OR partnership* OR participat* OR conferenc* OR meeting* OR mediat*) AND ((child* OR tee…	1,309
18	(child protection) OR (social work) OR (child advocacy) OR (child welfare)	17,479
17	((child* OR teenage* OR adolescen* OR toddler*) AND ((“child abuse”) OR (abuse* OR neglect* OR maltreat*))) AND (“group conferenc*” OR “group decision” OR “team conferenc*” OR “team decision*"…	13
16	((child* OR teenage* OR adolescen* OR toddler*) AND ((“child abuse”) OR (abuse* OR neglect* OR maltreat*))) AND (“family group” OR “family decision*” OR “family conferenc*” OR “family meeting"…	73
15	"child protection conferenc*” OR “child protection meeting*” OR “child protection mediation"	0
14	engag* OR involv* OR partnership* OR participat* OR conferenc* OR meeting* OR mediat*	34,274
13	child protection	5,224
12	social work	10,278
11	child advocacy	882
10	child welfare	4,721
9	(child* OR teenage* OR adolescen* OR toddler*) AND ((“child abuse”) OR (abuse* OR neglect* OR maltreat*))	7,870
8	child* OR teenage* OR adolescen* OR toddler*	38,074
6	(“child abuse”) OR (abuse* OR neglect* OR maltreat*)	10,444
5	"child abuse"	4,840
4	abuse* OR neglect* OR maltreat*	10,444
3	"group conferenc*” OR “group decision” OR “team conferenc*” OR “team decision*” OR “case meeting” OR "case planning” OR “planning meeting” OR "consensus‐based decision‐making" OR “consensus based de…	57
2	famil* OR parent* OR caregiver* OR guardian*	42,876
1	“family group” OR “family decision*” OR “family conferenc*” OR “family meeting” OR “family unity” OR “family team” OR “family centred” OR “family centered” OR FGC OR FGDM	370

### CINAHL database using the EbscoHost platform: Search Strategy (20 July 2016)

**Table:**

Set#	Searched for	Results
S23	S20 OR S22	19
S22	S19 AND S21	18
S21	"random* control* trial*” OR “random* control* stud*"	53,928
S20	S14 OR S15 OR S17 OR S18	9
S19	S14 OR S15 OR S17 OR S18	817
S18	S2 AND S13	12
S17	S2 AND S7 AND S12 AND S16	718
S16	S8 OR S9 OR S10 OR S11	32,402
S15	S2 AND S3 AND S7	11
S14	S1 AND S7	115
S13	"child protection conference*” OR “child protection meeting*” OR “child protection mediation"	16
S12	engag* OR involv* OR partnership* OR participat* OR conferenc* OR meeting* OR mediat*	353,053
S11	"child protection"	1,374
S10	(MH “Social Work+”)	11,654
S9	(MH “Child Advocacy”)	1,789
S8	(MH “Child Welfare+”)	22,103
S7	S4 OR S5	20,269
S6	S4 OR S5	70,483
S5	(MH “Child Abuse+”)	12,258
S4	abuse* OR neglect* OR maltreat*	70,378
S3	"group conferenc*” OR “group decision” OR “team conferenc*” OR “team decision*” OR “case meeting” OR “case planning” OR “planning meeting” OR "consensus‐based decision‐making" OR “consensus based decision making” OR “consensus based decisionmaking"	313
S2	(famil* OR parent* OR caregiver* OR guardian*) OR ((MH “Parents+”) OR (MH “Mothers+”) OR (MH “Fathers+”) OR (MH “Family+”))	288,940
S1	“family group” OR “family decision*” OR “family conferenc*” OR “family meeting” OR “family unity” OR “family team” OR "family centred” OR “family centered” OR FGC OR FGDM OR MH “Decision Making, Family"	9,300

### ERIC using the EbscoHost platform: Search Strategy (20 July 2016)

**Table:**

Set#	Searched for	Results
S22	S20 AND S21	2
S21	“random* control* trial*” OR “random* control* stud*"	1,635
S20	S15 OR S16 OR S18 OR S19	752
S19	S2 AND S14	3
S18	S2 AND S8 AND S13 AND S17	666
S17	S9 OR S10 OR S11 OR S12	12,884
S16	S2 AND S3 AND S8	14
S15	S1 AND S8	94
S14	"child protection conference*” OR “child protection meeting*” OR “child protection mediation"	3
S13	engag* OR involv* OR partnership* OR participat* OR conferenc* OR meeting* OR mediat*	445,573
S12	"child protection"	751
S11	DE “Social Work"	5,387
S10	DE “Child Advocacy"	2,109
S9	DE “Child Welfare"	6,010
S8	S6 AND S7	18,797
S7	child* or adolescen* OR teenage* OR infan* OR toddler* OR "pre‐school" OR “school age"	367,380
S6	S4 OR S5	33,794
S5	DE “Child Abuse"	7,823
S4	abuse* OR neglect* OR maltreat*	33,794
S3	"group conferenc*” OR “group decision” OR “team conferenc*” OR “team decision*” OR “case meeting” OR “case planning” OR “planning meeting” OR "consensus‐based decision‐making" OR “consensus based decision making” OR “consensus based decisionmaking"	719
S2	(DE “Family Involvement” OR DE “Parent Participation” OR DE “Family (Sociological Unit” OR DE “Parents” OR DE "Mothers” OR DE “Fathers”) OR (famil* OR parent* OR caregiver* OR guardian*)	219,348
S1	“family group” OR “family decision*” OR “family conferenc*” OR “family meeting” OR “family unity” OR “family team” OR “family centred” OR “family centered” OR FGC OR FGDM	1,304

### SocIndex using the EbscoHost platform: Search Strategy (20 July 2016)

**Table:**

Set#	Searched for	Results
S25	S20 AND S24	2
S24	S22 OR S23	3,700
S23	DE “RANDOMIZED controlled trials"	1,398
S22	"random* control* trial*” OR “random* control* stud*"	3,700
S21	S1 AND S3 AND S5	22
S20	S15 OR S16 OR S18 OR S19	309
S19	S2 AND S14	37
S18	S2 AND S8 AND S13 AND S17	257
S17	S9 OR S10 OR S11 OR S12	21,527
S16	S2 AND S3 AND S8	7
S15	S1 AND S8	25
S14	TI (“child protection conference*” OR “child protection meeting*” OR “child protection mediation”) OR SU (“child protection conference*” OR “child protection meeting*” OR “child protection mediation”) OR AB (“child protection conference*” OR “child protection meeting*” OR “child protection mediation”) OR KW (“child protection conference*” OR “child protection meeting*” OR “child protection mediation”)	53
S13	TI (engage* OR involve* OR partnership* OR participat* OR conferenc* OR meeting* OR mediat*) OR SU (engage* OR involve* OR partnership* OR participat* OR conferenc* OR meeting* OR mediat*) OR AB (engage* OR involve* OR partnership* OR participat* OR conferenc* OR meeting* OR mediat*) OR KW (engage* OR involve* OR partnership* OR participat* OR conferenc* OR meeting* OR mediat*)	332,473
S12	TI “child protection” OR SU “child protection” OR AB “child protection” OR KW “child protection” OR DE “CHILD protection services"	6,310
S11	DE “SOCIAL case work” OR DE “CONFIDENTIAL communications ‐‐ Social case work” OR DE “FAMILY social work” OR DE “SOCIAL case work reporting” OR DE “SOCIAL case work with children” OR DE “SOCIAL case work with teenagers” OR DE “SOCIAL case work with youth” OR DE “SOCIAL case work with children” OR DE “SOCIAL case work with teenagers"	4,760
S10	DE “CHILD advocacy (Law)"	174
S9	DE “CHILD welfare"	13,722
S8	S6 AND S7	6,028
S7	DE “CHILDREN” OR DE “TEENAGERS"	47,068
S6	S4 OR S5	120,134
S5	DE “CHILD abuse” OR DE “ABUSED children"	14,291
S4	TI (abuse OR neglect OR maltreat*) OR SU (abuse OR neglect OR maltreat*) OR AB (abuse OR neglect OR maltreat*) OR KW (abuse OR neglect OR maltreat*)	119,890
S3	TI (“group conferenc*” OR “group decision*” OR “team conferenc*” OR “team decision*” OR “case meeting*” OR “case planning” OR “planning meeting*” OR "consensus‐based decision‐making" OR “consensus based decision making” OR “consensus based decisionmaking”) OR SU (“group conferenc*” OR “group decision*” OR “team conferenc*” OR “team decision*” OR “case meeting*” OR “case planning” OR “planning meeting*” OR "consensus‐based decision‐making" OR “consensus based decision making” OR “consensus based decisionmaking”) OR AB (“group conferenc*” OR “group decision*” OR “team conferenc*” OR “team decision*” OR “case meeting*” OR “case planning” OR “planning meeting*” OR "consensus‐based decision‐making" OR “consensus based decision making” OR “consensus based decisionmaking”) OR KW (“group conferenc*” OR “group decision*” OR “team conferenc*” OR “team decision*” OR “case meeting*” OR “case planning” OR “planning meeting*” OR "consensus‐based decision‐making" OR “consensus based decision making” OR “consensus based decisionmaking”) AND DE "GROUP decision making"	2,128
S2	(famil* OR parent* OR caregiver* OR guardian*) OR TI (famil* OR parent* OR caregiver* OR guardian*) OR SU (famil* OR parent* OR caregiver* OR guardian*) OR KW (famil* OR parent* OR caregiver* OR guardian*) OR (famil* OR parent* OR caregiver* OR guardian*) OR DE “FAMILIES” OR DE “PARENTS” DE “CHILD caregivers” OR DE “MOTHERS” OR DE “FATHERS"	380,981
S1	TI (“family group” OR “family decision” OR “family conferenc*” OR “family meeting*” OR “family unity” OR “family team” OR “family centered” OR “family centred” OR FGC OR FGM) OR SU (“family group” OR “family decision” OR “family conferenc*” OR “family meeting*” OR “family unity” OR “family team” OR “family centered” OR “family centred” OR FGC OR FGM) OR AB (“family group” OR “family decision” OR “family conferenc*” OR “family meeting*” OR “family unity” OR “family team” OR “family centered” OR “family centred” OR OR FGC OR FGM) OR KW (“family group” OR “family decision” OR “family conferenc*” OR “family meeting*” OR “family unity” OR “family team” OR “family centered” OR “family centred” OR FGC OR FGM)	

### Medline using the OVID platform: Search Strategy (14 July 2016)

**Table:**

Set#	Searched for	Results
1	(family group or family decision* or family conferenc* or family meeting or family unity or family team or family centred or family centered or FGC or FGDM).tw.	4,484
2	(famil* or parent* or caregiver* or guardian*).tw.	1,145,310
3	(group conference* or group decision* or team conference* or team decision* or case meeting or case planning or planning meeting or consensus‐based decision‐making or consensus based decision making or consensus based decisionmaking).tw.	857
4	(abuse* or neglect* or maltreat*).tw.	150,328
5	exp child abuse/	26,297
6	4 or 5	159,169
7	limit 6 to (“all infant (birth to 23 months)” or “all child (0 to 18 years)”)	52,419
8	exp child welfare/	29,089
9	exp child advocacy/	3,904
10	exp social work/	16,289
11	child protection.tw.	1,412
12	(engag* or involv* or partnership or participat* or conferenc* or meeting* or mediat*).tw.	3,173,662
13	(child protection conference* or child protection meeting* or child protection mediat*).tw.	2
14	1 and 7	69
15	2 and 3 and 7	14
16	8 or 9 or 10 or 11	45,148
17	2 and 7 and 12 and 16	611
18	2 and 13	2
19	14 or 15 or 17 or 18	675
20	randomized controlled trial.pt.	423,885
21	randomized controlled trials as topic.sh.	108,073
22	controlled clinical trial.pt.	91,206
23	clinical trial.pt.	503,254
24	exp clinical trials as topic/	298,692
25	random allocation.sh.	87,750
26	double‐blind method.sh.	137,485
27	single‐blind method.sh.	22,335
28	((singl* or doubl* or trebl* or tripl*) adj25 (blind* or mask*)).ti,ab.	149,749
29	random*.ti,ab.	848,092
30	research design.sh.	90,163
31	comparative study.sh.	1,755,856
32	exp evaluation studies/	221,160
33	follow up studies.sh.	554,365
34	prospective studies.sh.	423,852
35	(control* or prospectiv*).ti,ab.	3,402,454
36	20 or 21 or 22 or 23 or 24 or 25 or 26 or 27 or 28 or 29 or 30 or 31 or 32 or 33 or 34 or 35	6,024,873
37	19 and 36	186

### EMBASE searched via the OVID platform (20 July 2016)

**Table:**

Set#	Searched for	Results
1	(family group or family decision* or family conferenc* or family meeting or family unity or family team or family centred or family centered or FGC or FGDM).tw.	5,745
2	(famil* or parent* or caregiver* or guardian*).tw.	1,458,664
3	(group conference* or group decision* or team conference* or team decision* or case meeting or case planning or planning meeting or consensus‐based decision‐making or consensus based decision making or consensus based decisionmaking).tw.	1,173
4	(abuse* or neglect* or maltreat*).tw.	196,683
5	limit 4 to (infant <to one year> or child <unspecified age> or preschool child <1 to 6 years> or school child <7 to 12 years> or adolescent <13 to 17 years>)	38,160
6	exp child abuse/	32,083
7	exp child welfare/	16,960
8	exp child advocacy/	3,171
9	child protection.tw.	1,827
10	exp social work/	23,230
11	exp community care/	107,829
12	(engag* or involv* or partnership or participat* or conferenc* or meeting* or mediat*).tw.	4,013,539
13	(child protection conference* or child protection meeting* or child protection mediat*).tw.	5
14	5 or 6	56,614
15	1 and 14	89
16	2 and 3 and 14	18
17	7 or 8 or 9 or 10 or 11	147,249
18	2 and 12 and 14 and 17	780
19	2 and 13	2
20	15 or 16 or 18 or 19	867
21	limit 20 to (clinical trial or randomized controlled trial or controlled clinical trial or phase 1 clinical trial or phase 2 clinical trial or phase 3 clinical trial or phase 4 clinical trial)	44
22	randomized controlled trial.tw.	58,941
23	clinical trial.tw.	138,976
24	((singl* or doubl* or trebl* or tripl*) adj25 (blind* or mask*)).ti,ab.	202,492
25	random*.ti,ab.	1,116,806
26	exp comparative study/	1,142,867
27	exp prospective study/	344,044
28	(control* or prospectiv*).ti,ab.	4,525,323
29	randomised controlled trial.tw.	18,866
30	22 or 23 or 24 or 25 or 26 or 27 or 28 or 29	6,033,580
31	20 and 30	203
32	21 or 31	210

### PsycINFO searched using the OVID platform (14 July 2016): search strategy

**Table:**

Set#	Searched for	Results
1	(family group or family decision* or family conferenc* or family meeting or family unity or family team or family centred or family centered or FGC or FGDM).mp.	4,670
2	(famil* or parent* or caregiver* or guardian*).mp.	550,505
3	(group conference* or group decision* or team conferenc* or team decision* or case meeting or case planning or planning meeting or consensus‐based decision‐making or consensus based decision making or consensus based decisionmaking).mp.	4,346
4	(abuse* or neglect* or maltreat*).mp.	176,931
5	exp child abuse/	25,163
6	exp abandonment/	437
7	exp child neglect/	3,513
8	exp emotional abuse/	2,189
9	exp physical abuse/	5,316
10	exp sexual abuse/	24,450
11	exp verbal abuse/	429
12	4 or 5 or 6 or 7 or 8 or 9 or 10 or 11	182,530
13	limit 12 to (100 childhood <birth to age 12 yrs> or 120 neonatal <birth to age 1 mo> or 140 infancy <2 to 23 mo> or 160 preschool age <age 2 to 5 yrs> or 180 school age <age 6 to 12 yrs> or 200 adolescence <age 13 to 17 yrs>)	41,953
14	exp child welfare/	7,212
15	exp social casework/	15,762
16	exp protective services/	2,350
17	exp social services/	38,290
18	child protection.mp.	3,322
19	(engag* or involv* or partnership or participat* or conferenc* or meeting* or mediat*).mp.	864,677
20	(child protection conference$ or child protection meeting$ or child protection mediation).mp.	41
21	1 and 13	114
22	2 and 3 and 13	24
23	14 or 15 or 16 or 17 or 18	58,153
24	2 and 13 and 19 and 23	764
25	2 and 20	26
26	21 or 22 or 24 or 25	889
27	1 and 18	48
28	26 or 27	930
29	limit 28 to (“0200 clinical case study” or “0400 empirical study” or “0410 experimental replication” or “0430 followup study” or “0450 longitudinal study” or “0451 prospective study” or “0453 retrospective study” or 1200 meta analysis or 1800 quantitative study or “2000 treatment outcome/clinical trial”)	559
30	(randomised controlled trial or randomized controlled trial).mp.	13,858
31	(random* adj25 allocat*).ti,ab.	3,717
32	exp clinical trials/	9,670
33	exp treatment effectiveness evaluation/	20,513
34	((singl* or doubl* or trebl* or tripl*) adj25 (blind* or mask*)).ti,ab.	23,197
35	(random* or control* or prospectiv*).ti,ab.	682,112
36	30 or 31 or 32 or 33 or 34 or 35	702,054
37	28 and 36	132
38	29 or 37	581

### Evidence‐Based Medicine Reviews was searched via the OVID platform on 14 July 2016

Evidence‐Based Medicine Reviews combines Cochrane Central Register of Controlled Trials; Cochrane Database of Systematic Reviews; Cochrane Database of Methodology Reviews, The Database of Abstracts of Reviews of Effectiveness (DARE); Health Technology Assessments; NHS Economic Evaluation Database; and the ACP Journal Club from the American College of Physicians.

**Table:**

Set#	Searched for	Results
1	(family group or family decision* or family conferenc* or family meeting or family unity or family team or family centred or family centered or FGC or FGDM).mp.	347
		288
		59
2	(famil* or parent* or caregiver* or guardian*).mp.	42,329
		38,274
		4,055
3	(group conference* or group decision* or team conferenc* or team decision* or case meeting or case planning or planning meeting or consensus‐based decision‐making or consensus based decision making or consensus based decisionmaking).mp.	114
		82
		32
4	(abuse* or neglect* or maltreat*).mp.	7,911
		7,079
		832
5	(infant or baby or toddler or preschooler or child or school age* or teen* or adolescent).mp.	151,697
		148,176
		3,521
6	(child welfare or social casework or social work* or protective services or social services or child protection).mp.	1,510
		1,165
		345
7	(engag* or involv* or partnership or participat* or conferenc* or meeting* or mediat*).mp.	129,357
		120,706
		8,651
8	(child protection conference* or child protection meeting* or child protection mediat*).mp.	0
	EBM Reviews—Cochrane Central Register of Controlled Trials <June 2016>	0
	EBM Reviews ‐ Cochrane Database of Systematic Reviews <2005 to July 13, 2016>	0
9	1 and 4 and 5	25
		7
		18
10	2 and 3 and 4 and 5	6
		3
		3
11	2 and 4 and 5 and 6 and 7	126
		48
		78
12	9 or 10 or 11	142
		54
		88
